# Comprehensive genome-wide analysis of genetic loci and candidate genes associated with litter traits in purebred Berkshire pigs of Korea

**DOI:** 10.5713/ab.24.0046

**Published:** 2024-08-18

**Authors:** Jun Park

**Affiliations:** 1 Department of Animal Biotechnology, Jeonbuk National University, Jeonju 54896, Korea; 2 Dasan Pig Breeding Co., Namwon, 55716, Korea

**Keywords:** Berkshire, Candidate Genes, Genome-wide Association Study, Litter Size, Reproductive Traits

## Abstract

**Objective:**

The objective of this study was to identify genomic regions and candidate genes associated with the total number of piglets born (TNB), number of piglets born alive (NBA), and total number of stillbirths (TNS) in Berkshire pigs.

**Methods:**

This study used a total of 11,228 records and 2,843 single-nucleotide polymorphism (SNP) data obtained from Illumina porcine 60 K and 80 K chips. The estimated genomic breeding values (GEBVs) and SNP effects were estimated using weighted single-step genomic BLUP (WssGBLUP).

**Results:**

The heritabilities of the TNB, NBA, and TNS were determined using single-step genomic best linear unbiased prediction (ssGBLUP). The heritability estimates were 0.13, 0.12, and 0.015 for TNB, NBA, and TNS, respectively. When comparing the accuracy of breeding value estimates, the results using pedigree-based BLUP (PBLUP) were 0.58, 0.60, and 0.31 for TNB, NBA, and TNS, respectively. In contrast, the accuracy increased to 0.67, 0.66, and 0.42 for TNB, NBA, and TNS, respectively, when using WssGBLUP, specifically in the last three iterations. The results of weighted single-step genome-wide association studies (WssGWAS) showed that the highest variance explained for each trait was predominantly located in the *Sus scrofa* chromosome 5 (SSC5) region. Specifically, the variance exceeded 4% for TNB, 3% for NBA, and 6% for TNS. Within the SSC5 region (12.26 to 12.76 Mb), which exhibited the highest variance for TNB, 20 SNPs were identified, and five candidate genes were identified: *TIMP3*, *SYN3*, *FBXO7*, *BPIFC*, and *RTCB*.

**Conclusion:**

The identified SNP markers for TNB, NBA, and TNS were expected to provide valuable information for genetic improvement as an understanding of their expression and genetic architecture in Berkshire pigs. With the accumulation of more phenotype and SNP data in the future, it is anticipated that more effective SNP markers will be identified.

## INTRODUCTION

Livestock reproductive traits are economically significant but predominantly sex-specific (such as sperm quality in males and fertility in females), and most involve complex genetic mechanisms with low heritability. Consequently, genetic improvement in these traits is particularly challenging compared with other economically relevant traits. Reproductive efficiency in sows has a substantial impact on the profitability of pig farming, which relies on factors such as litter size, gestation length, and farrowing intervals. These factors are significant in enhancing farm earnings, even with relatively low heritability. Notably, total piglets born, and live births play a critical role in evaluating reproductive efficiency and its influence on farm profits. However, pursuing larger litters may inadvertently increase piglet mortality and reduce gestation periods, as suggested by earlier studies. In addition, stillbirth rates may vary depending on the breed and breeding type. Enhancing litter size remains the primary breeding objective for many well-established breeding systems and organizations that have devoted decades to rigorous selection and breeding efforts [1]. Although traditional breeding strategies have yielded genetic improvements in these traits, the need for molecular breeding methods, such as genomic selection, has intensified to achieve faster rates of improvement.

Globally, there are more than 1,000 pig breeds; however, since the late 20th century, a relatively small number of breeds have been used for commercial pig production because of intensive selective breeding and genetic improvement. This strategy has led to improved reproductive capabilities, growth rates, carcass yields, muscle growth, and intramuscular fat content. In Korea, the swine sector predominantly employs a three-breed cross (Yorkshire, Landrace, and Duroc) in a pyramidal breeding structure to produce commercial pigs. These crossbreeds, which are globally prevalent, have shifted toward specialized, high-quality pork for enhanced profitability. The Berkshire breed, known for its superior meat quality [2,3], has been considered for breeding to improve meat characteristics [4]. Many previous studies have highlighted differences in fatty acid composition among pig breeds. Berkshires, for instance, have significantly higher saturated fatty acid and lower monounsaturated fatty acid content than Duroc and Landrace [5]. Differences in fatty acids, such as palmitoleic acid, oleic acid, linoleic acid, and linolenic acid, between Pulawska and Polish Landrace contribute to the superior meat quality of native species [6]. Variations in meat quality characteristics and fatty acid composition are attributable to breed differences, which impact consumer-recognized meat attributes. However, Berkshires pose rearing challenges because of their smaller litters and lower piglet survival rates [7], making litter size enhancement crucial for leveraging their meat quality attributes.

Genome-wide association studies (GWAS) have revolu tionized molecular breeding and genetics, particularly in identifying and analyzing economically important traits in livestock. These studies have led to the discovery of multiple candidate genes and significant genetic markers, often revealing complex interactions at the same genomic locus. Such complexities, inherent in quantitative trait studies influenced by multiple genes and environmental factors, present challenges in detecting quantitative trait loci (QTLs) and mapping accuracy [8]. The advent of high-density single-nucleotide polymorphism (SNP) panels has greatly improved the precision of QTL mapping and candidate gene identification. These methods provide more accurate analyses of trait heritability than conventional pedigree assessments [9]. In the realm of GWAS, the primary techniques used are single-SNP GWAS and the Bayesian approach. The single-SNP method treats each SNP as a distinct fixed effect, which acknowledges variations within population groups. Conversely, the Bayesian approach assesses all SNPs simultaneously [10]. However, in Korea, there is a substantial disparity between the availability of phenotype and genotype data, with far fewer animals having complete sets of both types of data. This data gap restricts the effective application of these methods, primarily because of the need to compute pseudo-phenotypes, such as deregressed breeding values [11]. To address these challenges, a single-step GWAS (ssGWAS) was developed. This method leverages the genomic enhanced breeding values (GEBVs), calculated via single-step genomic best linear unbiased prediction (ssGBLUP), to estimate the impacts on individual SNPs. Its premise of uniform variance across all markers, however, may limit its utility in traits significantly influenced by major QTLs. To circumvent these limitations, the weighted ssGWAS (WssGWAS) methodology was introduced. This advanced approach variably weights SNP effects according to their relevance to the trait under investigation, thus enhancing the accuracy of QTL identification. The WssGWAS integrates GEBVs derived from phenotypes, genotypes, and pedigree information, addressing unequal variances among SNPs, and promoting more precise SNP effect estimations [10,12]. This method proves especially advantageous for traits heavily affected by significant QTL effects, particularly in situations where phenotype and genotype data are scarce [13]. The implementation of WssGWAS begins with the calculation of the inverse of the realized relationship matrix (*H*^−1^), which incorporates all available pedigree and genotype information. This matrix is used within the ssGBLUP framework to compute GEBV for each animal, and these values are then used to assess the impact of individual SNP effects. Subsequently, these effects were analyzed to determine the proportion of genetic variance accounted for by sequential SNP groups or windows. Although WssGWAS does not directly extract SNP effects from the model and lacks mechanisms for evaluating statistical test uncertainties, it provides critical insights into the most significant SNP windows based on explained genetic variance. This method is highly recognized in QTL detection research, despite not facilitating formal significance testing. Our study adopted the WssGWAS approach because it effectively combines phenotypic, genotypic, and pedigree data, thus eliminating the need to generate pseudo-phenotypes for genotyped animals. This strategy not only assigns variable weights to SNPs based on their significance but also surpasses the simplistic assumptions of the GBLUP infinitesimal model, thereby enhancing the precision of SNP effect estimations. Moreover, the methodology of assessing consecutive SNP windows due to linkage disequilibrium (LD) proves more effective in pinpointing QTL regions than the analysis of individual SNPs. Overall, advancements in genomic analysis through WssGWAS have profoundly enriched our understanding of the genetic architecture of economically important traits in livestock, significantly influencing breeding strategies and enabling more informed selection and breeding decisions to optimize desirable traits in livestock populations.

This study builds upon our establishment of a Berkshire breed lineage and initial research on meat quality genetic parameters [14]. We conducted a preliminary study on pH, a key meat quality trait, in the Berkshire breed. Beginning with an investigation of meat quality traits, we conducted this study to improve litter size in Berkshire pigs. We identified genetic regions and candidate genes linked to litter size in domestic Berkshire pigs using WssGWAS.

## MATERIALS AND METHODS

### Animals and phenotypes

Phenotypic data were sourced from D Farm, a commercial pig farm in Korea, which has been implementing a proprietary breeding program since 2003, initially with pigs imported from the US. Presently, the farm exclusively uses its own breeding plan to produce purebred and candidate pigs, foregoing the need for further imports. D Farm’s Berkshire pigs are recognized as a unique single breed both in Korea and internationally by the United Nations Food and Agriculture Organization (FAO) and the domestic animal diversity information system (DAD-IS). This study analyzed 11,228 reproductive records, focusing on the total number of piglets born (TNB), number of piglets born alive (NBA), and total number of stillbirths (TNS) (Supplementary Table S1). TNB refers to the TNB per parturition, including stillbirths and mummies. NBA is the count of piglets, excluding mummies and stillbirths, from the total born. TNS represents the sum of mummies and stillbirths.

### Single-nucleotide polymorphism data and quality control

Genotypic data were gathered from 2,076 samples using the Illumina 60K beadchip and from 773 samples using the Illumina 80K beadchip. Quality control procedures were implemented via PLINK [15], with exclusions for SNPs with unknown positions, those on sex chromosomes, a call rate below 0.90, a minor allele frequency under 0.01, or a significant departure from Hardy–Weinberg equilibrium (p<10^−6^). Parentage verification was performed using SEEKPARENTF90 [16] with a 10% threshold for resolving paternity discrepancies. After the reconciliation of genotyped animals and the alignment of genotypic identification with phenotypic and pedigree information, 2,843 animals were selected for the GWAS. The 60 K data were imputed to the 80 K standard using the latter as a reference, and phasing was performed using SHAPEIT4 [17], which is a fast and accurate method for haplotype estimation that uses a PBWT-based approach to select informative conditioning haplotypes. Imputation was then conducted using IMPUTE5 [18], which assumes phased samples with no missing alleles. The concluding dataset for the analysis contained 53,812 markers.

### Statistical analyses

Genetic parameters for TNB, NBA, and TNS were estimated using the average information restricted maximum likelihood (AIREML) method. Two distinct approaches were utilized: pedigree-based BLUP (PBLUP) and ssGBLUP. This statistical model facilitates the partitioning of observed phenotypic variances into genetic and environmental components, thereby enabling the estimation of heritability and genetic correlations between traits. The PBLUP approach incorporates pedigree information to estimate genetic effects, whereas the ssGBLUP method integrates both pedigree and genomic information, potentially enhancing the precision of genetic parameter estimates. Each trait was analyzed using a single-trait animal model. The model equation is as follows (1):


y=Xb+Za+Wpe+e

Where *y* is the vector of phenotypic observations; *b* is the vector of fixed effects (birth year-season and parity); *a* is the vector of additive genetic effects; *pe* is the vector of permanent environmental effects; *e* is the vector of residuals; and *X*, *Z*, and *W* are the incidence matrices of *b*, *a*, and *pe*, respectively. Heritability was estimated as $h^{2}=\frac{\sigma_{a}^{2}}{\sigma_{a}^{2}+\sigma_{pe}^{2}+\sigma_{e}^{2}}$, where $\sigma_{a}^{2},\;\sigma_{pe}^{2}$, and $\sigma_{e}^{2}$ were additive genetic, permanent environmental, and residual variance, respectively.

Furthermore, GEBVs were calculated using the ssGBLUP approach, and marker effects were derived from these GEBVs. In contrast to the conventional BLUP approach, ssGBLUP substitutes the inverse of the pedigree relationship matrix (*A*^−1^) with the inverse of the combined matrix *H*^−1^, which incorporated both the pedigree and genomic relationship matrices [19]. The *H*^−1^ can be represented as follows (2):


$$H^{-1}=A^{-1}+\left[\begin{array}{cc} 0 & 0\\ 0 & G^{-1}-A_{22}^{-1} \end{array}\right]$$

where $A_{22}^{-1}$ is the inverse of the numerator relationship matrix for genotyped pigs, and *G* refers to the genomic relationship matrix [20]. *G* is presented below:


$$G=\frac{ZDZ^{\prime }}{\Sigma_{i=1}^{M}2p_{i}(1-p_{i})}$$

where *Z* is a matrix of gene content adjusted for allele frequencies (*0*, *1*, or *2* for *AA*, *Aa*, and *aa*, respectively), *D* is a diagonal matrix of weights for SNP variances (initially *D* = *I*), *M* is the number of SNPs, and *p_i_* is the minor allele frequency of *i*th SNP. Estimates of the SNP effects and weights for WssGWAS were obtained according to the following steps [10]:

i) First step (t = 1): *D* = *I*; *G*_(_*_t_*_)_ = *D*_(_*_t_*_)_ *Z*′*λ*, where $\lambda =\frac{1}{\Sigma_{i=1}^{M}\;2p_{i}(1-p_{i})}$;
ii) Calculate GEBVs;
iii) Convert GEBVs to SNP effects $\hat{u}=\lambda D_{\left(t\right)}Z^{\prime }G_{\left(t\right)}^{-1}{\hat{a}}_{g}$, where *â_g_* was the GEBV of the animal that was also genotyped.
iv) Calculate the weight for each SNP: $d_{i(t+1)}={\hat{u}}_{i(t)}^{2}2p_{i}(1-p_{i})$, where *i* was the *i*th SNP;
v) Normalize the SNP weights to keep the total genetic variance constant:
$$D_{\left(t+1\right)}=\frac{tr(D_{\left(1\right)})}{tr(D_{\left(t+1\right)})}D_{\left(t+1\right)}$$
vi) *G*_(_*_t_*_+1)_ = *ZD*_(_*_t_*_+1)_ *Z′λ* was calculated.
vii) *t* = *t*+1 and loop to step 2.

The procedure comprised three iterative cycles to refine the accuracy of GEBV [21,22]. In each cycle, the weights assigned to SNPs were updated (step 4 and 5), and these updated weights were then used for several key steps: constructing G matrices (step 6), recalculating the GEBV (step 2), and estimating the effects of SNPs (step 3). After updating the SNP weights, we computed the proportion of genetic variance accounted for by each successive group of SNPs, which are termed as the *i*th SNP windows [22]. For this study, SNPs within the 0.52 Mb range (a window size determined by the decay of LD in the studied population) were grouped together. The percentage of genetic variance explained by each of these 0.52 Mb SNP windows was then calculated as follows:


$$\frac{Var(a_{i})}{\sigma_{a}^{2}}=\frac{Var(\Sigma_{j=1}^{x}Z_{j}{\hat{u}}_{j})}{\sigma_{a}^{2}}\times 100$$

where *a_i_* is the genetic value of the *i*th SNP window that consisted of a region of consecutive SNPs located within 0.52 Mb, *Z_j_* was the vector of gene content of the *j*th SNP for all individuals, and *û_j_* was the effect of the *j*th SNP within the *i*th window. To visualize the distribution of these SNP windows, Manhattan plots were generated using R software and the CMplot package [23,24]. The procedures described above were implemented iteratively using the BLUPF90 software suite [25].

### Linkage disequilibrium decay estimation and identification of candidate genes

To evaluate the average LD decay across the Berkshire pig genome, we calculated the squared correlation (*r*^2^) between alleles using PLINK v1.9 [15], with a set window size of 1 Mb. This analysis revealed that the average LD decay distance is approximately 520 kb, which is the point at which the *r*^2^ value drops to 0.2. Using this LD decay distance, we then calculated the genetic variance using 0.52 Mb windows.

To identify significant SNP windows impacting TNB and NBA traits, we established a threshold of 1.56%. This threshold, informed by literature reviews [26,27] and the anticipated contribution of SNP windows to these traits [28], aligns with the WssGWAS criteria [29]. SNP windows explaining 1.56% or more of the total genetic variance were marked as significant, consistent with the expected 50-fold variance contribution of individual SNP windows (0.031% ×50 = 1.56%).

In studying tick resistance, we identified top windows containing QTL based on their genetic variance contribution, employing a similar methodology. The POSTGSF90 tool was used on a dataset of 2,843 animals and 53,812 SNPs to locate these top windows.

After pinpointing significant windows, candidate genes within these regions were explored using the Ensembl *Sus scrofa* 11.1 database (https://www.ensembl.org/biomart). This comprehensive pig genomic database allowed us to align significant SNP windows with the genome, facilitating the identification of potential genes affecting the traits under investigation. These findings offer valuable insights for further genetic analysis and could inform targeted improvements in breeding programs.

## RESULTS AND DISCUSSION

In our study, we compared the heritability of productive traits using the PBLUP and ssGBLUP methods (Table 1). In this study, we analyzed the heritability and standard error estimates for TNB, NBA, and TNS using the PBLUP and ssGBLUP methods. The estimates obtained with PBLUP were 0.12 (0.021), 0.14 (0.021), and 0.013 (0.010) for TNB, NBA, and TNS, respectively. In addition, the ssGBLUP method provided estimates of 0.13 (0.018), 0.12 (0.017), and 0.015 (0.009) for the same traits. Although the differences in heritability estimates were not statistically significant, the standard errors were consistently lower with ssGBLUP. These findings align with research indicating that integrating genomic data with pedigree information theoretically enhances the accuracy of estimated parameters [30].

In terms of trait accuracy, PBLUP demonstrated the lowest accuracy, whereas the accuracy of WssGBLUP improved with additional iterations (Table 2). Compared with PBLUP, the accuracy of WssGBLUP with weights assigned after three iterations increased by approximately 15% for TNB, 10% for NBA, and 35% for TNS. These findings suggest that genomic data usage can lead to more precise breeding value estimations for traits with low heritability, like TNS, thereby potentially enhancing breeding efficiency.

**Figure 1:**
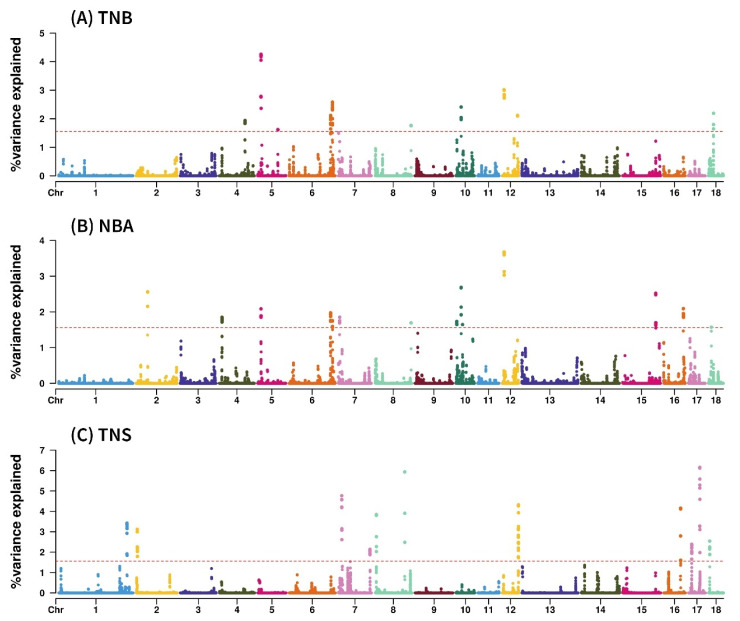
Manhattan plots of a genome-wide association study of litter traits in Berkshire pigs. (A) Total number of piglets born (TNB), (B) number of piglets born alive (NBA), and (C) total number of stillbirths (TNS). Each dot represents one single-nucleotide polymorphism window of 0.52 Mb. On the y-axis is the percentage of genetic variance explained by the windows.

Molecular breeding identifies candidate genes linked to quantitative traits with complex genetic architectures. In pig breeding, reproductive performance, particularly litter size, directly affects farm profitability. Our study employed WssGWAS to estimate the genetic variance explained by 0.52 Mb windows for TNB, NBA, and TNS (Figure 1). In the GWAS results, the regions with the highest explained genetic variance were over 4% in the *Sus scrofa* chromosome 5 (SSC5) region for TNB (Table 3), over 6% in the SSC 12 region for NBA (Table 4), and over 6% in the SSC 17 region for TNS (Table 5).

Moreover, for TNB, regions exceeding the threshold in cluded SSC 4, 6, 8, 10, 12, and 18. In the SSC12 region (7.28 to 7.78 Mb) with the highest variance for NBA, 31 SNPs and two candidate genes (*RPL38*, *ENSSSCG00000046071*) were identified. In addition, for NBA, regions surpassing the threshold were SSC 2, 4, 5, 7, 8, 10, 15, 16, and 18. For TNS, the SSC17 region (42.21 to 42.70 Mb) displayed the highest variance, with 12 SNPs and three candidate genes (*ENSSSCG 00000052330*, *ENSSSCG00000059132*, *DHX35*) identified. Threshold-exceeding regions for TNS were SSC 1, 2, 7, 8, 12, 16, and 18. Common candidate genes for TNB and NBA were identified because of their high correlation (*TIMP3*, *SYN3*, *FBXO7*, *BPIFC* in SSC 5, *OSBPL9* in SSC 6, *COPS4, THAP9, SEC31A, SCD5, TMEM150C* in SSC 8, *TLR5, SUSD4, CVPN2* in SSC 10, *RPL38, ENSSSCG00000046071* in SSC 12), but none were common with TNS in this study. Our research identified SNPs exceeding 1.56% in variance, explaining 149 for TNB, 187 for NBA, and 182 (Supplementary Table S2 to S4). These findings offer significant insights into the genetic basis of litter traits in pigs and present potential genetic markers for breeding programs. Generally, the heritability of litter traits is lower than that of growth traits. In this study, the heritability of TNB and NBA were 0.1 in the ssGBLUP analysis. Given TNS’s very low heritability, using genomic data in evaluating traits with low genetic influence becomes crucial, underscoring the importance of this research. Considering the minimal genetic influence, our study identified regions associated with litter size using WssGBLUP and WssGWAS. The analysis results indicate that SSC 8, SSC 12, and SSC 18 consistently exceeded the threshold for each trait and are therefore considered the most critical regions in this study.

We analyzed the genotypes of SNPs in regions demonstrat ing the highest genetic variance for each trait (Supplementary Figure S1 to S3). In TNB, the region at 12 Mb on SSC5 showed an additive variance of 4.26%. SNP analysis within this area revealed that in heterozygous individuals, TNB did not exceed nine, with the marker ASGA0024492 displaying the highest TNB in the minor homozygous (GG) group at 9.05, 0.36 higher than that in the major homozygous (AA) group. For NBA, the 7 Mb region on SSC12 had an additive variance of 3.68%. The marker ASGA0084859 in this region showed the largest litter size in the major homozygous group (GG) at 9.50, 1.75 more than that in the minor homozygous group (AA). For TNS, the 42 Mb region on SSC17 presented an additive variance of 6.16%. The marker WU_10.2_17_47838187 in this region indicated the lowest TNS in the major homozygous group (GG) at 0.90, 0.12 less than in the minor homozygous group (AA), and 0.15 less than that in the heterozygous group. Although the differences were not substantial, the average TNS being less than one is significant because it could directly impact farm productivity, suggesting its potential as an important marker for use.

The locus on SSC5, which encompasses genes *TIMP3*, S*YN3*, *FBXO7*, *BPIFC*, and *RTCB*, exhibited notable variance in relation to TNB. The *TIMP* gene family, including *TIMP1* through *TIMP4*, serves as a physiological inhibitor of matrix metalloproteinases (MMPs). *TIMP3* is distinguished by its strong affinity for proteoglycans in the extracellular matrix (ECM) and its broad substrate specificity, impacting MMPs, ADAMs (a disintegrin and metalloproteinases), and ADAMTSs (ADAM with thrombospondin motifs) [31]. Furthermore, despite stable *TIMP3* transcription levels in porcine ovarian cysts, an increase at the protein level implies post-transcriptional or post-translational adjustments, possibly linked to cyst development [32]. *TIMP3*, an inhibitor of ECM component degradation, showed increased expression in the testicular tissue of Duroc pigs with a high DNA Fragmentation Index (DFI), suggesting a significant role in regulating sperm DNA integrity and ECM stability [33]. *FBXO7*, part of the *SKP1-CUL1-F-box* (*SCF*) *E3 ubiquitin-protein ligase complex*, is critical for substrate identification, phosphorylation-dependent ubiquitination, and subsequent proteasomal degradation of target proteins [34,35]. It also aids in the assembly of cyclin D–Cdk6 complexes, interacting with D-type cyclins and Cdk6, and is expressed in various organs and tissues [36]. The *TRAP/SSR complex*, consisting of *TRAPα/SSR1*, *TRAPβ/SSR2*, *TRAPγ/SSR3*, and *TRAPδ/SSR4*, facilitates protein translocation, particularly for proteins with signal peptides that poorly interact with the Sec61 complex [37]. In pancreatic β-cells under high-glucose conditions, the upregulation of *TRAP* subunit mRNAs has been observed, indicating their importance in β-cell functionality [38]. A deficiency in the *TRAPα/SSR1* gene is linked to disrupted preproinsulin translocation and decreased insulin storage in pancreatic β-cells [39]. The Sushi domain-containing 4 (*SUSD4*) gene, which is critical in neurodevelopment, is associated with neurodevelopmental disorders and immune system interactions, including its correlation with intestinal microbiota composition in pigs [40,41].

The locus on SSC12, which encompasses genes *RPL38* and *ENSSSCG00000046071*, showed significant variance related to NBA. Although these genes have been reported as potential candidate genes associated with productive traits such as average daily gain and days to 105 kg (AGE) in Yorkshire pigs, no direct associations with swine reproductive traits have been reported [42]. The *SOX6* gene on SSC2, which displayed the second-highest variance explained in NBA, has been identified as a candidate gene associated with NBA [43]. It plays a critical role in regulating fetal muscle development [44], which may indirectly influence the survival of newborn pigs. In addition, toll-like receptors (TLRs), including *TLR5*, are pivotal in innate immunity by regulating antimicrobial responses in mucosal tissues. Their expression in the endometrium and placenta of pigs is crucial for controlling mucosal immune responses to support the establishment and maintenance of pregnancy [45].

The locus on SSC17, which encompasses genes *ENSSSCG 00000052330*, *ENSSSCG00000059132*, and *DHX35*, exhibited notable variance in relation to TNS. The *ENPEP* gene, located in the SSC8 region, has been reported to play a significant role in regulating blood flow and angiogenesis in the endometrium, with direct implications for fetal wellbeing during late pregnancy [46]. Ataxin-1 (*ATXN1*), a candidate gene for body weight in pigs, is involved in cytoskeleton organization and microtubule dynamics. Higher expression of *myostatin* (*MSTN*) in growing animals compared with transitional piglets [47], with variations among pig breeds, has been noted. Given the reported role of *ATXN1* in regulating fetal skin development [48], these genes could be linked to stillbirth rates in pigs.

## CONCLUSION

Our research has effectively used the WssGWAS and WssGBLUP methodologies to enhance our understanding of genetic influences on productive traits in pigs, particularly focusing on reproductive performance. Through WssGWAS, we identified critical genomic regions and candidate genes, such as those in the SSC5 region, that significantly impact the variance in traits like TNB, NBA, and TNS. Our comparative analysis using PBLUP and WssGBLUP demonstrated that WssGBLUP offers more accurate heritability estimates by integrating both pedigree and genomic information. This precision is especially valuable for traits with low heritability, such as TNS, underscoring the need to incorporate genomic data into breeding value estimations to improve breeding efficiency. In addition, analyzing consecutive SNP windows in GWAS proved more effective than focusing on individual SNPs, enhancing our ability to identify additional influential regions and candidate genes.

To build upon these findings, we will conduct further re search to validate and refine the genomic techniques used. This includes expanding our data collection to encompass larger and more diverse pig populations, implementing identified genomic markers in controlled breeding experiments, and cross-validating these markers across various breeds and conditions. We also plan to apply advanced genomic techniques to develop new traits, enhance disease resistance, and improve feed efficiency. These initiatives are crucial for broadening the scope of our genetic research and are expected to significantly improve the health and efficiency of pig populations.

In conclusion, our study underscores the essential role of sophisticated genomic techniques in deciphering the complex genetic architecture of productive traits in pigs. These methods have established a foundation for the development of more precise and efficient breeding strategies that enhance farm productivity and profitability. By extending these methodologies to include research on disease resistance, feed efficiency, and the development of new traits, we aim to address crucial yet challenging traits due to their low heritability, further advancing pig breeding research.

## SUPPLEMENTARY MATERIAL

Supplementary file is available from: https://doi.org/10.5713/ab.24.0046

**Supplementary Table S1**. Basic statistics for litter traits of Korean Berkshire pigs

**Supplementary Table S2**. Summary of GWAS with the significant 0.52 Mb windows that were associated with the total number of piglets born (TNB) in Korean 3 Berkshire pigs

**Supplementary Table S3**. Summary of GWAS with the significant 0.52 Mb windows that were associated with the number of piglets born alive (NBA) in Korean 5 Berkshire pigs

**Supplementary Table S4**. Summary of GWAS with the significant 0.52 Mb windows that were associated with the total number of stillbirths (TNS) in Korean Berkshire 7 pigs

**Supplementary Figure S1**. The phenotypic change according to the genotype of the markers that showed the highest genetic variance explained in TNB.

**Supplementary Figure S2**. The phenotypic change according to the genotype of the markers that showed the highest genetic variance explained in NBA.

**Supplementary Figure S3**. The phenotypic change according to the genotype of the markers that showed the highest genetic variance explained in TNS.

## Figures and Tables

**Table 1:** Variance components and heritabilities for litter traits

Traits	Method	$\sigma_{a}^{2}$	$\;\sigma_{pe}^{2}$	$\;\sigma_{e}^{2}$	*h*^2^ (SE)
TNB	PBLUP	0.86403	0.70290	5.4841	0.12 (0.021)
	ssGBLUP	0.90049	0.68482	5.4853	0.13 (0.018)
NBA	PBLUP	0.85656	0.40453	4.75030	0.14 (0.021)
	ssGBLUP	0.71397	0.51030	4.75300	0.12 (0.017)
TNS	PBLUP	0.01717	0.08573	1.35620	0.013 (0.010)
	ssGBLUP	0.02017	0.08328	1.25330	0.015 (0.009)

$\sigma_{a}^{2}$, additive genetic; $\;\sigma_{pe}^{2}$, permanent environment; $\;\sigma_{e}^{2}$, residual variances; *h*^2^ (SE), heritability and standard error; PBLUP, pedigree based best linear unbiased prediction; ssGBLUP, single-step genomic BLUP; TNB, total number of piglets born; NBA, number of piglets born alive; TNS, total number of stillbirths.

**Table 2:** Comparison of the accuracy of PBLUP and WssGBLUP according to the number of iterations

Trait	PBLUP	WssGBUP		

		Iteration 1	Iteration 2	Iteration 3
TNB	0.58	0.62	0.63	0.67
NBA	0.60	0.62	0.63	0.66
TNS	0.31	0.33	0.35	0.42

PBLUP, pedigree based best linear unbiased prediction; WssGBLUP, weighted single-step genomic BLUP in each iteration; TNB, total number of piglets born; NBA, number of piglets born alive; TNS, total number of stillbirths.

**Table 3:** Significant SNPs associated with the total number of piglets born (TNB) in Korean Berkshire pigs

SSC	Position (Mb)	gVar (%)^1)^	Nsnp^2)^	Candidate genes
4	95.60–96.11	1.94	17	*IL6R, ATP8B2, HAX1, UBAP2L, CFA141, ENSSSCG00000006556, NUP210L*
5	12.26–12.76	4.26	20	*TIMP3, SYN3, FBXO7, BPIFC, RTCB*
	74.65–75.13	1.62	18	*PUS7L, TWF1, TMEM117*
6	153.91–154.42	2.12	30	*MYSM1, TACSTD2, OMA1*
	160.47–160.99	2.58	13	*RAB3B, NRDC, ENSSSCG00000058239, OSBPL9*
8	135.27–135.79	1.77	28	*COPS4, THAP9, SEC31A, SCD5, TMEM150C*
10	19.51–20.00	2.41	19	*TLR5, SUSD4, CAPN8*
12	7.01–7.52	3.01	31	*RPL38, ENSSSCG00000046071*
	56.87–57.37	2.12	17	*ENSSSCG00000038836*
18	19.10–19.55	2.19	14	*NRF1*

SNPs, single-nucleotide polymorphisms; SSC, *Sus scrofa* chromosome.

1) Percentage of genetic variance explained by 0.52 Mb.

2) Number of SNPs belonging to the position (Mb).

**Table 4:** Significant SNPs associated with the number of piglets born alive (NBA) in Korean Berkshire pigs

SSC	Position (Mb)	gVar (%)^1)^	Nsnp^2)^	Candidate genes
2	43.24–43.74	2.56	17	*SOX6*
4	10.39–10.91	1.85	23	*ASAP1, ENSSSCG00000005959*
5	12.27–12.77	2.08	20	*TIMP3, SYN3, FBXO7, BPIFC*
6	153.63–154.15	1.98	27	*ENSSSCG00000056712, ENSSSCG00000053758*
	160.54–161.05	1.75	12	*OSBPL9*
7	5.09–5.61	1.85	28	*SSR1, CAGE1, RIOK1, ENSSSCG00000043207, DSP, SNRNP48, BMP6*
8	135.67–136.18	1.69	31	*ENSSSCG00000009240, COPS4, THAP9, SEC31A, SCD5, TMEM150C*
10	3.50–3.95	1.73	13	*ENSSSCG00000057470, ENSSSCG00000053245*
	19.51–20.00	2.69	19	*TLR5, SUSD4, CAPN2*
	24.80–25.30	1.64	13	*KDM5B*
12	7.28–7.78	3.68	31	*RPL38, ENSSSCG00000046071*
15	122.54–123.05	2.52	20	*ENSSSCG00000060048, ENSSSCG00000059125, EPHA4*
16	72.81–73.29	2.09	20	*SEMA5A*
18	11.25–11.72	1.57	10	-

SNPs, single-nucleotide polymorphisms; SSC, *Sus scrofa* chromosome.

1) Percentage of genetic variance explained by 0.52 Mb.

2) Number of SNPs belonging to the position (Mb).

**Table 5:** Significant SNPs associated with the total number of stillbirths (TNS) trait in Korean Berkshire pigs

SSC	Position (Mb)	gVar (%)^1)^	Nsnp^2)^	Candidate genes
1	251.78–252.30	3.42	21	*MUSK, ENSSSCG00000005457, ECPAS, ENSSSCG00000044924, OR2K2, ECPAS*
2	4.09–4.61	3.12	22	*TPCN2, IGHMBP2, CPT1A, PPP6R3, LRP5*
7	11.97–12.49	4.77	22	*ENSSSCG00000061595, ENSSSCG00000057253, ATXN1, GMPR*
	116.36–116.88	2.14	22	*ENSSSCG00000052470, DICER1, ENSSSCG00000042615, ENSSSCG00000057191*
8	6.81–7.33	3.84	25	*CLNK, ENSSSCG00000019781*
	111.76–112.28	5.93	10	*ENSSSCG00000052805, ENSSSCG00000048102, ENPEP*
12	59.68–60.19	4.32	16	*NCOR1, SPECC1, AKAP10, ULK2, ADORA2B, SPECC1*
16	62.00–62.49	4.16	13	*GABRA6, ENSSSCG00000041732*
17	12.15–12.67	2.38	19	*ENSSSCG00000007004, ENSSSCG00000044124, ENSSSCG00000050111, ENSSSCG00000053036, ENSSSCG00000050358*
	42.21–42.70	6.16	12	*ENSSSCG00000052330, ENSSSCG00000059132, DHX35*
18	4.15–4.66	2.54	27	*DPP6, ENSSSCG00000042794*

SNPs, single-nucleotide polymorphisms; SSC, *Sus scrofa* chromosome.

1) Percentage of genetic variance explained by 0.52 Mb.

2) Number of SNPs belonging to the position (Mb).
