# Supplementary material for: Comprehensive genome-wide analysis of genetic loci and candidate genes associated with litter traits in purebred Berkshire pigs of Korea
Source: Anim Biosci. 2024 Aug 18;37(10):1702–11. doi: 10.5713/ab.24.0046 (PMC11366516; doi:10.5713/ab.24.0046)
Supplement: Supplementary file 1 [file ab-24-0046-Supplementary-Table-1.pdf]

SUPPLEMENTARY MATERIAL

Supplemntary Table S1 Basic statistics for litter traits of Korean Berkshire pigs.
Supplemntary Table S2 Summary of GWAS with the significant 0.52 Mb windows that were associated with the total number of piglets born (TNB) in Korean Berkshire pigs.
Supplemntary Table S3 Summary of GWAS with the significant 0.52 Mb windows that were associated with the number of piglets born alive (NBA) in Korean Berkshire pigs
Supplemntary Table S4 Summary of GWAS with the significant 0.52 Mb windows that were associated with the total number of stillbirths (TNS) in Korean Berkshire pigs
Supplemntary Figure S1 The phenotypic change according to the genotype of the markers that showed the highest genetic variance explained in TNB
Supplemntary Figure S2 The phenotypic change according to the genotype of the markers that showed the highest genetic variance explained in NBA
Supplemntary Figure S3 The phenotypic change according to the genotype of the markers that showed the highest genetic variance explained in TNS

Table S1 Basic statistics for litter traits of Korean Berkshire pigs.

Traits[1]	No. of records	Mean	SD[2]	Minimum	Maximum
TNB	11,228	8.95	2.69	2	22
NBA	11,228	7.92	2.53	0	18
TNS	11,228	1.03	1.26	0	14

[1]total number of piglets born (TNB), the number of piglets born alive (NBA), total number of stillbirths (TNS); [2]SD: Strandard deviation

Table S2 Summary of GWAS with the significant 0.52 Mb windows that were associated with the total number of piglets born (TNB) in Korean Berkshire pigs.

SNP	rsid	chr	Position	Variant	SNP effect	gVar(%)[1]	Gene annotation[2]
H3GA0013593	rs80954318	4	95331300	intron	6.98128E-09	1.8	IL6R
H3GA0013596	rs80909580	4	95369253	intron	2.08063E-10	1.9	IL6R
ALGA0026990	rs81382415	4	95430762	synonymous	1.67196E-06	1.9	ATP8B2
MARC0105246	rs1110880965	4	95470082	intron	-8.30154E-08	1.9	HAX1
ALGA0026994	rs1109800220	4	95485304	intron	4.04223E-06	1.9	UBAP2L
ASGA0021116	rs81382427	4	95509695	intron	4.37379E-15	1.9	UBAP2L
DIAS0000761	rs346114470	4	95533483	missense	-2.879E-10	1.9	CFAP141
ASGA0021118	rs81382433	4	95557016	intron	1.57495E-05	1.9	ENSSSCG00000006556
MARC0027501	rs80977488	4	95601325	intron	0.003482441	1.9	NUP210L
ALGA0027004	rs3471618871	4	95669839	intron	-0.03380295	1.9	NUP210L
WU_10.2_5_11843429	rs336985696	5	12081150	intergenic	-4.7398E-10	2.8	ssc-mir-9790 (52632), TIMP3 (161431)
