# Supplementary material for: Comprehensive genome-wide analysis of genetic loci and candidate genes associated with litter traits in purebred Berkshire pigs of Korea
Source: Anim Biosci. 2024 Aug 18;37(10):1702–11. doi: 10.5713/ab.24.0046 (PMC11366516; doi:10.5713/ab.24.0046)
Supplement: Supplementary file 2 [file ab-24-0046-Supplementary-Table-2.pdf]

SUPPLEMENTARY MATERIAL

Supplemntary Table S1 Basic statistics for litter traits of Korean Berkshire pigs.
Supplemntary Table S2 Summary of GWAS with the significant 0.52 Mb windows that were associated with the total number of piglets born (TNB) in Korean Berkshire pigs.
Supplemntary Table S3 Summary of GWAS with the significant 0.52 Mb windows that were associated with the number of piglets born alive (NBA) in Korean Berkshire pigs
Supplemntary Table S4 Summary of GWAS with the significant 0.52 Mb windows that were associated with the total number of stillbirths (TNS) in Korean Berkshire pigs
Supplemntary Figure S1 The phenotypic change according to the genotype of the markers that showed the highest genetic variance explained in TNB
Supplemntary Figure S2 The phenotypic change according to the genotype of the markers that showed the highest genetic variance explained in NBA
Supplemntary Figure S3 The phenotypic change according to the genotype of the markers that showed the highest genetic variance explained in TNS

Table S1 Basic statistics for litter traits of Korean Berkshire pigs.

Traits[1]	No. of records	Mean	SD[2]	Minimum	Maximum
TNB	11,228	8.95	2.69	2	22
NBA	11,228	7.92	2.53	0	18
TNS	11,228	1.03	1.26	0	14

[1]total number of piglets born (TNB), the number of piglets born alive (NBA), total number of stillbirths (TNS); [2]SD: Strandard deviation

Table S2 Summary of GWAS with the significant 0.52 Mb windows that were associated with the total number of piglets born (TNB) in Korean Berkshire pigs.

SNP	rsid	chr	Position	Variant	SNP effect	gVar(%)[1]	Gene annotation[2]
H3GA0013593	rs80954318	4	95331300	intron	6.98128E-09	1.8	IL6R
H3GA0013596	rs80909580	4	95369253	intron	2.08063E-10	1.9	IL6R
ALGA0026990	rs81382415	4	95430762	synonymous	1.67196E-06	1.9	ATP8B2
MARC0105246	rs1110880965	4	95470082	intron	-8.30154E-08	1.9	HAX1
ALGA0026994	rs1109800220	4	95485304	intron	4.04223E-06	1.9	UBAP2L
ASGA0021116	rs81382427	4	95509695	intron	4.37379E-15	1.9	UBAP2L
DIAS0000761	rs346114470	4	95533483	missense	-2.879E-10	1.9	CFAP141
ASGA0021118	rs81382433	4	95557016	intron	1.57495E-05	1.9	ENSSSCG00000006556
MARC0027501	rs80977488	4	95601325	intron	0.003482441	1.9	NUP210L
ALGA0027004	rs3471618871	4	95669839	intron	-0.03380295	1.9	NUP210L
WU_10.2_5_11843429	rs336985696	5	12081150	intergenic	-4.7398E-10	2.8	ssc-mir-9790 (52632), TIMP3 (161431)

WU_10.2_5_11812749	rs318311504	5	12113071	intergenic	-9.03172E-10	2.8	ssc-mir-9790 (84553), TIMP3 (129510)
WU_10.2_5_11786856	rs335156436	5	12138941	intergenic	-2.85702E-10	2.8	ssc-mir-9790 (110423), TIMP3 (103640)
WU_10.2_5_11768030	rs331901219	5	12157755	intergenic	-0.000132706	2.8	ssc-mir-9790 (129237), TIMP3 (84826)
ALGA0030559	rs80953600	5	12158272	intergenic	-0.005433828	2.8	ssc-mir-9790 (129754), TIMP3 (84309)
WU_10.2_5_11963093	rs339691066	5	12186789	3_prime_UTR	3.01442E-07	2.8	TIMP3
INRA0018475	rs329285273	5	12204810	intron	-1.94785E-08	2.8	TIMP3
H3GA0015679	rs80924517	5	12226072	intron	7.67983E-07	2.8	TIMP3
WU_10.2_5_2468497	rs335297714	5	12265777	intergenic	-0.008796267	4.3	TIMP3 (79959), SYN3 (234299)
WU_10.2_5_88710499	rs326845351	5	12267943	intergenic	-1.68881E-08	4.1	TIMP3 (82125), SYN3 (232133)
ASGA0024487	rs81387465	5	12306038	intergenic	-0.000161242	4.2	TIMP3 (120220), SYN3 (194038)
WU_10.2_5_12166660	rs339872670	5	12372257	intergenic	3.83899E-05	4.2	TIMP3 (186439), SYN3 (127819)
ALGA0030566	rs81387478	5	12398727	intergenic	-1.69982E-08	4.2	TIMP3 (212909), SYN3 (101349)
ASGA0024488	rs81387482	5	12452062	intergenic	-6.96834E-09	4.2	TIMP3 (266244), SYN3 (48014)
WU_10.2_5_12259315	rs342151864	5	12473396	upstream	0.000179405	4.2	TIMP3 (287578), SYN3 (26680)
H3GA0015682	rs80849107	5	12487593	intron	-1.60238E-08	4.2	SYN3
INRA0018490	rs324200582	5	12510020	intron	-7.69021E-08	4.2	FBXO7
H3GA0015686	rs80795165	5	12544917	intron	-8.24148E-05	4.2	BPIFC
ASGA0024492	rs80989679	5	12567885	intron	-0.04491719	4.2	BPIFC
ASGA0024492	rs80989679	5	12567885	intron	-0.04491719	4.2	BPIFC (25619), BPIFC (11069)
DIAS0003292	rs80815903	5	12596918	synonymous	-0.08753973	2.4	RTCB
DIAS0003292	rs80815903	5	12596918	synonymous	-0.08753973	2.4	RTCB (16624), RTCB (16934)
WU_10.2_5_77458038	rs324971369	5	74645799	intron	-6.57516E-05	1.6	ADAMTS20
ASGA0026341	rs80859829	5	74704457	intergenic	6.40983E-08	1.6	ADAMTS20 (201341), PUS7L (129971)
ALGA0032936	rs80891783	5	74721790	intergenic	-3.34132E-07	1.6	ADAMTS20 (218674), PUS7L (112638)
H3GA0016866	rs80841456	5	74743829	intergenic	3.50036E-10	1.6	ADAMTS20 (240713), PUS7L (90599)
H3GA0016864	rs80886325	5	74761472	intergenic	3.0488E-12	1.6	ADAMTS20 (258356), PUS7L (72956)
ALGA0032933	rs80873221	5	74783135	intergenic	3.83405E-13	1.6	ADAMTS20 (280019), PUS7L (51293)

DIAS0004288	rs80898295	5	74806259	missense	3.11627E-08	1.6	PUS7L
WU_10.2_5_77683641	rs326683948	5	74871138	intron	6.13455E-14	1.6	TWF1
ALGA0112619	rs81341240	5	74891309	intron	2.30876E-12	1.6	TWF1
ALGA0117169	rs81346881	5	74924814	intron	0.000252938	1.6	TMEM117
ALGA0032946	rs80897897	5	74981466	intron	-0.02990466	1.6	TMEM117
ALGA0037340	rs81393036	6	153576745	upstream	4.72502E-06	2	ENSSSCG00000061831 (7479), ENSSSCG00000052447 (11120)
ALGA0037343	rs81393043	6	153597317	non_coding_transcript_exon	-5.85011E-05	2	ENSSSCG00000056712
ALGA0037343	rs81393043	6	153597317	non_coding_transcript_exon	-5.85011E-05	2	ENSSSCG00000056712 (4312), ENSSSCG00000056712 (4272)
WU_10.2_6_141178397	rs332119045	6	153633196	non_coding_transcript_exon	-0.005316871	2	ENSSSCG00000053758
ASGA0029872	rs81393079	6	153644923	intergenic	-5.62839E-07	1.8	ENSSSCG00000053758 (14870), ENSSSCG00000056388 (39679)
WU_10.2_6_141192870	rs338944012	6	153648450	intergenic	-0.000905295	1.8	ENSSSCG00000053758 (18397), ENSSSCG00000056388 (36152)
H3GA0019067	rs81393069	6	153668879	intergenic	-0.001184369	1.8	ENSSSCG00000053758 (38826), ENSSSCG00000056388 (15723)
MARC0057158	rs81246878	6	153675300	upstream	-3.65967E-05	1.8	ENSSSCG00000053758 (45247), ENSSSCG00000056388 (9302)
ALGA0037350	rs81393063	6	153702154	intergenic	-0.00024595	1.8	JUN (16472), MYSM1 (93381)
WU_10.2_6_141248398	rs319529181	6	153703701	intergenic	0.02180048	1.8	JUN (18019), MYSM1 (91834)
ALGA0037357	rs80892726	6	153740906	intergenic	-6.71541E-10	1.6	JUN (55224), MYSM1 (54629)
ASGA0029874	rs81393087	6	153754973	upstream	2.37258E-09	1.6	JUN (69291), MYSM1 (40562)
WU_10.2_6_141309639	rs334967097	6	153767092	intron	1.71885E-06	1.6	MYSM1
WU_10.2_6_141383152	rs328746765	6	153789144	intron	-0.001186055	2	MYSM1
WU_10.2_6_141383152	rs328746765	6	153789144	intron	-0.001186055	2	MYSM1 (32962), MYSM1 (6391)
MARC0033192	rs81227426	6	153823337	intergenic	3.61809E-06	2.1	MYSM1 (67155), TACSTD2 (38155)
ASGA0099800	rs81320394	6	153833562	intergenic	-0.005551801	2.1	MYSM1 (77380), TACSTD2 (27930)
ALGA0108422	rs81336326	6	153848968	intergenic	5.25214E-05	2.1	MYSM1 (92786), TACSTD2 (12524)
WU_10.2_6_141454842	rs320246646	6	153860889	3_prime_UTR	1.89666E-06	2.1	TACSTD2
WU_10.2_6_141499839	rs333017624	6	153905462	intron	-0.03525992	2.1	OMA1
ASGA0030100	rs81393717	6	160220231	intron	-3.60366E-05	1.8	RAB3B

H3GA0019195	rs81393714	6	160251796	3_prime_UTR	-0.000169383	1.8	RAB3B
ASGA0030114	rs81393734	6	160327199	3_prime_UTR	-7.75316E-08	1.8	NRDC
ASGA0030114	rs81393734	6	160327199	3_prime_UTR	-7.75316E-08	1.8	ENSSSCG00000058239
MARC0029221	rs81224030	6	160361778	intron	-1.44451E-06	1.8	NRDC
MARC0029221	rs81224030	6	160361778	intron	-1.44451E-06	1.8	MIR761 (25622), NRDC (26724)
WU_10.2_6_148093098	rs338350400	6	160390005	intron	2.57091E-06	2.4	OSBPL9
WU_10.2_6_148093098	rs338350400	6	160390005	intron	2.57091E-06	2.4	OSBPL9 (1328), OSBPL9 (176364)
WU_10.2_6_148132890	rs321816399	6	160429795	intron	-0.001360494	2.4	OSBPL9
WU_10.2_6_148158088	rs333191369	6	160454991	intron	7.54074E-05	2.3	OSBPL9
WU_10.2_6_148177389	rs343360489	6	160474293	intron	0.000815511	2.6	OSBPL9
WU_10.2_6_148209459	rs332227876	6	160506365	intron	0.002343421	2.6	OSBPL9
H3GA0019199	rs81393740	6	160535498	intron	0.005565753	2.5	OSBPL9
MARC0057560	rs81247226	6	160565896	intron	0.009697298	2.3	OSBPL9
H3GA0019200	rs81393756	6	160587182	intergenic	0.02606615	2	OSBPL9 (198505), ENSSSCG00000059473 (33459)
ASGA0040427	rs81405706	8	135274760	3_prime_UTR	3.66211E-06	1.8	ENSSSCG00000009240
ASGA0040427	rs81405706	8	135274760	3_prime_UTR	3.66211E-06	1.8	ENSSSCG00000009240 (24685), ENSSSCG00000009240 (4285)
ALGA0050145	rs81405710	8	135285532	intron	2.07845E-05	1.8	COPS4
WU_10.2_8_144693654	rs345777825	8	135398502	intron	1.11798E-11	1.8	THAP9
ALGA0117619	rs81347425	8	135427306	intron	3.2426E-06	1.8	SEC31A
ALGA0117619	rs81347425	8	135427306	intron	3.2426E-06	1.8	SEC31A (903), SEC31A (67815)
8_144744932	rs328673214	8	135449779	intron	4.53708E-06	1.8	SEC31A
WU_10.2_8_144771629	rs345296578	8	135476476	intron	4.26343E-06	1.8	SEC31A
WU_10.2_8_144771629	rs345296578	8	135476476	intron	4.26343E-06	1.8	SEC31A (50073), SEC31A (18645)
WU_10.2_8_144847860	rs334436239	8	135487158	intron	4.41681E-06	1.8	SEC31A
H3GA0052926	rs81336088	8	135491186	intron	5.67874E-06	1.8	SEC31A
WU_10.2_8_144898378	rs319261432	8	135537530	intron	0.000005701	1.8	SCD5
WU_10.2_8_144927686	rs320260240	8	135566615	intron	3.33286E-05	1.8	SCD5

ALGA0121391	rs81328969	8	135591552	intron	1.12855E-09	1.8	SCD5
H3GA0054370	rs81328785	8	135604963	intron	4.76261E-12	1.8	SCD5
DRGA0017418	rs81339189	8	135607348	intron	4.557E-11	1.8	SCD5
ALGA0109193	rs81337243	8	135629399	intron	4.61342E-11	1.8	SCD5
WU_10.2_8_145018548	rs338821365	8	135648560	downstream	2.26782E-10	1.8	ENSSSCG00000052420 (9892), ENSSSCG00000060705 (5196)
ASGA0082238	rs81330273	8	135665981	intergenic	2.84127E-06	1.8	ENSSSCG00000060705 (16178), U6 (54432)
ASGA0105760	rs81305967	8	135668712	intergenic	5.18215E-06	1.8	ENSSSCG00000060705 (18909), U6 (51701)
MARC0065298	rs81253423	8	135689152	intron	1.08384E-14	1.8	TMEM150C
ALGA0105830	rs339198846	8	135699278	intron	-0.001191077	1.8	TMEM150C
ASGA0092699	rs81311486	8	135719143	intron	3.08671E-05	1.8	TMEM150C
ASGA0102835	rs81476910	8	135728719	intron	2.98738E-05	1.8	TMEM150C
MARC0059365	rs81249045	8	135751832	intron	5.39231E-10	1.8	TMEM150C
ASGA0104181	rs81304104	8	135768303	downstream	0.0323693	1.8	U6 (47996), ENOPH1 (34085)
10007668	rs81218852	10	19391507	synonymous	-8.40999E-08	2.4	TLR5
H3GA0029533	rs81421707	10	19409070	intron	4.0851E-15	2.4	TLR5
CASI0009135	rs345690779	10	19411706	upstream	-2.77253E-07	2.4	TLR5 (24040), SUSD4 (140134)
ALGA0057679	rs81421752	10	19512266	intron	0.01312272	2.4	SUSD4
WU_10.2_10_22034045	rs344136854	10	19535825	intron	0.003421729	2	SUSD4
ASGA0046986	rs81421775	10	19572163	intergenic	-1.79565E-09	2	SUSD4 (150598), ENSSSCG00000010888 (14231)
WU_10.2_10_22164312	rs327591080	10	19604335	intergenic	-0.000983042	2	ENSSSCG00000010888 (19173), ENSSSCG00000054070 (54172)
WU_10.2_10_22123753	rs342419073	10	19645727	intergenic	-3.20411E-09	2	ENSSSCG00000010888 (60565), ENSSSCG00000054070 (12780)
MARC0010568	rs3474528732	10	19715055	intron	-4.20413E-06	2	CAPN8
ASGA0095681	rs81315152	10	19753037	intergenic	-6.7154E-08	2	CAPN8 (87182), CAPN2 (67209)
MARC0105091	rs3471802107	10	19780059	intron	0.02827226	2	CAPN2
ASGA0084454	rs81343376	12	6971812	intergenic	-1.93931E-07	3	RPL38 (86910), ENSSSCG00000046071 (647627)
MARC0075255	rs81260438	12	6982421	intergenic	4.32251E-08	3	RPL38 (97519), ENSSSCG00000046071 (637018)

M1GA0026980	rs81323860	12	6995769	intergenic	5.14487E-09	3	RPL38 (110867), ENSSSCG00000046071 (623670)
ALGA0103381	rs81478115	12	7006069	regulatory_region	2.25299E-08	3	RPL38 (121167), ENSSSCG00000046071 (613370)
ASGA0052681	rs81436220	12	7048858	intergenic	1.43673E-09	3	RPL38 (163956), ENSSSCG00000046071 (570581)
H3GA0033126	rs81436398	12	7067951	intergenic	-6.35279E-05	3	RPL38 (183049), ENSSSCG00000046071 (551488)
MARC0046612	rs81236893	12	7080205	intergenic	9.07607E-10	3	RPL38 (195303), ENSSSCG00000046071 (539234)
WU_10.2_12_7124825	rs345556034	12	7097729	intergenic	1.18092E-06	3	RPL38 (212827), ENSSSCG00000046071 (521710)
H3GA0033141	rs81436583	12	7127237	intergenic	-8.29972E-07	3	RPL38 (242335), ENSSSCG00000046071 (492202)
WU_10.2_12_7581758	rs337597858	12	7159210	intergenic	0.000181161	3	RPL38 (274308), ENSSSCG00000046071 (460229)
H3GA0033163	rs81436813	12	7188626	intergenic	-2.74889E-05	3	RPL38 (303724), ENSSSCG00000046071 (430813)
ALGA0064531	rs81436950	12	7225821	intergenic	-4.61117E-05	3	RPL38 (340919), ENSSSCG00000046071 (393618)
WU_10.2_12_7507773	rs326956108	12	7233173	intergenic	9.95661E-05	3	RPL38 (348271), ENSSSCG00000046071 (386266)
ASGA0052741	rs81437044	12	7247426	intergenic	-5.67751E-05	3	RPL38 (362524), ENSSSCG00000046071 (372013)
H3GA0033190	rs81437363	12	7283888	intergenic	1.06075E-07	3	RPL38 (398986), ENSSSCG00000046071 (335551)
DRGA0011569	rs81300895	12	7302134	intergenic	1.47152E-10	3	RPL38 (417232), ENSSSCG00000046071 (317305)
WU_10.2_12_7398515	rs81437611	12	7341458	intergenic	0.001955544	3	RPL38 (456556), ENSSSCG00000046071 (277981)
WU_10.2_12_7394362	rs325492649	12	7345606	intergenic	0.000310769	3	RPL38 (460704), ENSSSCG00000046071 (273833)
WU_10.2_12_7385966	rs318399475	12	7354001	intergenic	0.007386912	3	RPL38 (469099), ENSSSCG00000046071 (265438)
WU_10.2_12_7634052	rs338584089	12	7354388	intergenic	4.17818E-05	2.8	RPL38 (469486), ENSSSCG00000046071 (265051)
ASGA0090707	rs81309086	12	7371353	intergenic	0.003680733	2.8	RPL38 (486451), ENSSSCG00000046071 (248086)
ALGA0105065	rs81332319	12	7414272	intergenic	4.69927E-05	2.7	RPL38 (529370), ENSSSCG00000046071 (205167)
ASGA0095520	rs81314949	12	7418252	intergenic	6.55416E-05	2.7	RPL38 (533350), ENSSSCG00000046071 (201187)
ASGA0084859	rs335889358	12	7418384	regulatory_region	1.30564E-07	2.7	RPL38 (533482), ENSSSCG00000046071 (201055)
ASGA0083611	rs81338615	12	7420790	intergenic	1.69338E-12	2.7	RPL38 (535888), ENSSSCG00000046071 (198649)
H3GA0054071	rs81325851	12	7421785	intergenic	0.000198189	2.7	RPL38 (536883), ENSSSCG00000046071 (197654)
ASGA0092609	rs81311382	12	7458764	intergenic	1.38725E-07	2.7	RPL38 (573862), ENSSSCG00000046071 (160675)
M1GA0026970	rs81323725	12	7467740	intergenic	0.1205242	2.8	RPL38 (582838), ENSSSCG00000046071 (151699)
H3GA0034945	rs80819532	12	56869918	intron	-1.83799E-13	2.1	ENSSSCG00000038836

WU_10.2_12_59543325	rs341767777	12	56889946	intron	-1.27153E-17	2.1	ENSSSCG00000038836
M1GA0017107	rs80838117	12	56928205	upstream	0.05670551	2.1	ENSSSCG00000038836 (101682), MYOCD (101053)
ASGA0079091	rs81472357	18	18969597	downstream	-0.00339301	1.8	UBE2H (149956), ENSSSCG00000049100 (7597)
MARC0113374	rs81241389	18	19103011	intron	-0.01683279	2.2	NRF1
H3GA0053092	rs81338103	18	19109295	intron	-0.00863991	1.6	NRF1

[1]percentage of genetic variance explained by 0.52 Mb; [2]gene symbols when intronic or gene symbols (distance) adjacent to the marker when intergenic

Table S3 Summary of GWAS with the significant 0.52 Mb windows that were associated with the number of plglets born alive (NBA) in Korean Berkshire pigs.

**SNP**	**rsid**	**chr**	**Position**	**Variant**	**SNP effect**	**gVar(%)[1]**	**Gene annotation[2]**
DRGA0002978	rs81295479	2	43040842	intron	2.46565E-09	2.2	SOX6
H3GA0006696	rs81358422	2	43102031	intergenic	0.000320507	2.6	SOX6 (648350), ENSSSCG00000052631 (201717)
MARC0000439	rs81253718	2	43124329	intergenic	1.87211E-13	2.6	SOX6 (670648), ENSSSCG00000052631 (179419)
ALGA0013525	rs81358479	2	43191966	intergenic	5.39776E-10	2.6	SOX6 (738285), ENSSSCG00000052631 (111782)
WU_10.2_2_46373395	rs326649839	2	43228658	intergenic	-1.95202E-08	2.6	SOX6 (774977), ENSSSCG00000052631 (75090)
ALGA0013529	rs81358487	2	43237145	intergenic	2.17537E-05	2.6	SOX6 (783464), ENSSSCG00000052631 (66603)
MARC0041784	rs81234803	2	43294192	downstream	0.07713712	2.6	SOX6 (840511), ENSSSCG00000052631 (9556)
M1GA0005682	rs3476855545	4	10389912	intron	1.67557E-05	1.8	ASAP1
M1GA0005684	rs3473102742	4	10401987	intron	1.02125E-06	1.8	ASAP1
M1GA0005687	rs3472326377	4	10425479	intron	0.001525907	1.8	ASAP1
WU_10.2_4_10670656	rs340305872	4	10453726	downstream	0.001551937	1.8	ASAP1 (273968), ENSSSCG00000005959 (191547)
ALGA0023112	rs80797306	4	10470393	intergenic	0.001551937	1.8	ASAP1 (290635), ENSSSCG00000005959 (174880)
ALGA0023115	rs80974878	4	10487214	intron	0.001671321	1.8	ENSSSCG00000005959
H3GA0011950	rs80866732	4	10493687	intron	3.41872E-06	1.7	ENSSSCG00000005959
WU_10.2_4_10724127	rs342564651	4	10508063	intron	0.001085978	1.7	ENSSSCG00000005959
WU_10.2_4_10724127	rs342564651	4	10508063	intron	0.001085978	1.7	ENSSSCG00000005959 (31931), ENSSSCG00000005959 (137210)
DRGA0004454	rs80876417	4	10524007	intron	0.000982427	1.7	ENSSSCG00000005959
H3GA0011952	rs80880654	4	10547481	intron	-0.01713098	1.7	ENSSSCG00000005959
