# Supplementary material for: Comprehensive genome-wide analysis of genetic loci and candidate genes associated with litter traits in purebred Berkshire pigs of Korea
Source: Anim Biosci. 2024 Aug 18;37(10):1702–11. doi: 10.5713/ab.24.0046 (PMC11366516; doi:10.5713/ab.24.0046)
Supplement: Supplementary file 3 [file ab-24-0046-Supplementary-Table-3.pdf]

WU_10.2_12_59543325	rs341767777	12	56889946	intron	-1.27153E-17	2.1	ENSSSCG00000038836
M1GA0017107	rs80838117	12	56928205	upstream	0.05670551	2.1	ENSSSCG00000038836 (101682), MYOCD (101053)
ASGA0079091	rs81472357	18	18969597	downstream	-0.00339301	1.8	UBE2H (149956), ENSSSCG00000049100 (7597)
MARC0113374	rs81241389	18	19103011	intron	-0.01683279	2.2	NRF1
H3GA0053092	rs81338103	18	19109295	intron	-0.00863991	1.6	NRF1

[1]percentage of genetic variance explained by 0.52 Mb; [2]gene symbols when intronic or gene symbols (distance) adjacent to the marker when intergenic

Table S3 Summary of GWAS with the significant 0.52 Mb windows that were associated with the number of plglets born alive (NBA) in Korean Berkshire pigs.

SNP	rsid	chr	Position	Variant	SNP effect	gVar(%)[1]	Gene annotation[2]
DRGA0002978	rs81295479	2	43040842	intron	2.46565E-09	2.2	SOX6
H3GA0006696	rs81358422	2	43102031	intergenic	0.000320507	2.6	SOX6 (648350), ENSSSCG00000052631 (201717)
MARC0000439	rs81253718	2	43124329	intergenic	1.87211E-13	2.6	SOX6 (670648), ENSSSCG00000052631 (179419)
ALGA0013525	rs81358479	2	43191966	intergenic	5.39776E-10	2.6	SOX6 (738285), ENSSSCG00000052631 (111782)
WU_10.2_2_46373395	rs326649839	2	43228658	intergenic	-1.95202E-08	2.6	SOX6 (774977), ENSSSCG00000052631 (75090)
ALGA0013529	rs81358487	2	43237145	intergenic	2.17537E-05	2.6	SOX6 (783464), ENSSSCG00000052631 (66603)
MARC0041784	rs81234803	2	43294192	downstream	0.07713712	2.6	SOX6 (840511), ENSSSCG00000052631 (9556)
M1GA0005682	rs3476855545	4	10389912	intron	1.67557E-05	1.8	ASAP1
M1GA0005684	rs3473102742	4	10401987	intron	1.02125E-06	1.8	ASAP1
M1GA0005687	rs3472326377	4	10425479	intron	0.001525907	1.8	ASAP1
WU_10.2_4_10670656	rs340305872	4	10453726	downstream	0.001551937	1.8	ASAP1 (273968), ENSSSCG00000005959 (191547)
ALGA0023112	rs80797306	4	10470393	intergenic	0.001551937	1.8	ASAP1 (290635), ENSSSCG00000005959 (174880)
ALGA0023115	rs80974878	4	10487214	intron	0.001671321	1.8	ENSSSCG00000005959
H3GA0011950	rs80866732	4	10493687	intron	3.41872E-06	1.7	ENSSSCG00000005959
WU_10.2_4_10724127	rs342564651	4	10508063	intron	0.001085978	1.7	ENSSSCG00000005959
WU_10.2_4_10724127	rs342564651	4	10508063	intron	0.001085978	1.7	ENSSSCG00000005959 (31931), ENSSSCG00000005959 (137210)
DRGA0004454	rs80876417	4	10524007	intron	0.000982427	1.7	ENSSSCG00000005959
H3GA0011952	rs80880654	4	10547481	intron	-0.01713098	1.7	ENSSSCG00000005959

WU_10.2_5_2468497	rs335297714	5	12265777	intergenic	-0.01247181	2.1	TIMP3 (79959), SYN3 (234299)
WU_10.2_5_88710499	rs326845351	5	12267943	intergenic	3.91983E-08	1.8	TIMP3 (82125), SYN3 (232133)
ASGA0024487	rs81387465	5	12306038	intergenic	-0.000851405	1.9	TIMP3 (120220), SYN3 (194038)
WU_10.2_5_12166660	rs339872670	5	12372257	intergenic	-1.24088E-06	1.9	TIMP3 (186439), SYN3 (127819)
ALGA0030566	rs81387478	5	12398727	intergenic	6.58701E-08	1.9	TIMP3 (212909), SYN3 (101349)
ASGA0024488	rs81387482	5	12452062	intergenic	2.19254E-07	1.9	TIMP3 (266244), SYN3 (48014)
WU_10.2_5_12259315	rs342151864	5	12473396	upstream	-2.82082E-07	1.9	TIMP3 (287578), SYN3 (26680)
H3GA0015682	rs80849107	5	12487593	intron	1.01108E-07	1.9	SYN3
H3GA0015682	rs80849107	5	12487593	intron	1.01108E-07	1.9	SYN3 (13800), SYN3 (12483)
INRA0018490	rs324200582	5	12510020	intron	-3.61434E-07	1.9	FBXO7
H3GA0015686	rs80795165	5	12544917	intron	-0.000232642	1.9	BPIFC
ASGA0024492	rs80989679	5	12567885	intron	-0.04172075	1.9	BPIFC
ASGA0024492	rs80989679	5	12567885	intron	-0.04172075	1.9	BPIFC (25619), BPIFC (11069)
ALGA0037327	rs81393003	6	153507600	regulatory_region	-9.22856E-05	1.8	ENSSSCG00000060742 (14955), ENSSSCG00000055265 (18878)
ALGA0037331	rs81271871	6	153539867	upstream	0.001437153	1.8	ENSSSCG00000048387 (6026), ENSSSCG00000059728 (25661)
ALGA0037340	rs81393036	6	153576745	upstream	0.000112913	1.9	ENSSSCG00000061831 (7479), ENSSSCG00000052447 (11120)
ALGA0037343	rs81393043	6	153597317	non_coding_transcript_exon	-6.79478E-05	1.9	ENSSSCG00000056712
WU_10.2_6_141178397	rs332119045	6	153633196	non_coding_transcript_exon	-0.000655225	2	ENSSSCG00000053758
ASGA0029872	rs81393079	6	153644923	intergenic	-6.34489E-08	2	ENSSSCG00000053758 (14870), ENSSSCG00000056388 (39679)
WU_10.2_6_141192870	rs338944012	6	153648450	intergenic	-0.001400859	2	ENSSSCG00000053758 (18397), ENSSSCG00000056388 (36152)
H3GA0019067	rs81393069	6	153668879	intergenic	-0.001455317	1.9	ENSSSCG00000053758 (38826), ENSSSCG00000056388 (15723)
MARC0057158	rs81246878	6	153675300	upstream	3.02258E-06	1.9	ENSSSCG00000053758 (45247), ENSSSCG00000056388 (9302)
ALGA0037350	rs81393063	6	153702154	intergenic	-2.57948E-05	1.9	JUN (16472), MYSM1 (93381)
WU_10.2_6_141248398	rs319529181	6	153703701	intergenic	0.06760816	1.9	JUN (18019), MYSM1 (91834)
WU_10.2_6_148093098	rs338350400	6	160390005	intron	6.32793E-06	1.6	OSBPL9
WU_10.2_6_148132890	rs321816399	6	160429795	intron	-1.17752E-05	1.6	OSBPL9

WU_10.2_6_148158088	rs333191369	6	160454991	intron	-1.79602E-05	1.6	OSBPL9
WU_10.2_6_148177389	rs343360489	6	160474293	intron	5.52476E-05	1.7	OSBPL9
WU_10.2_6_148177389	rs343360489	6	160474293	intron	5.52476E-05	1.7	OSBPL9 (85616), OSBPL9 (92076)
WU_10.2_6_148209459	rs332227876	6	160506365	intron	-0.000306128	1.7	OSBPL9
H3GA0019199	rs81393740	6	160535498	intron	0.005828012	1.8	OSBPL9
MARC0057560	rs81247226	6	160565896	intron	0.003625864	1.6	OSBPL9
WU_10.2_7_4658272	rs80969350	7	4645765	intergenic	-0.00057687	1.7	ENSSSCG00000001021 (23912), SSR1 (40136)
ASGA0030712	rs80932912	7	4656246	intron	-0.000437539	1.7	SSR1
WU_10.2_7_4680039	rs323942012	7	4667522	intron	-0.00104853	1.7	SSR1
WU_10.2_7_4777306	rs319182788	7	4689809	upstream	2.11846E-07	1.7	SSR1 (36888), ENSSSCG00000062690 (5908)
DRGA0007051	rs80882694	7	4717571	intron	-1.30132E-13	1.7	CAGE1
DRGA0007051	rs80882694	7	4717571	intron	-1.30132E-13	1.7	CAGE1 (9811), CAGE1 (33102)
ALGA0038110	rs81397895	7	4748257	intron	2.84014E-10	1.7	CAGE1
ALGA0038112	rs80970321	7	4765310	intron	-1.26361E-08	1.7	RIOK1
WU_10.2_7_4885022	rs338024761	7	4779335	3_prime_UTR	-8.70562E-09	1.7	RIOK1
H3GA0019625	rs80964482	7	4785725	non_coding_transcript_exon	-8.70562E-09	1.7	ENSSSCG00000043207
H3GA0019625	rs80964482	7	4785725	non_coding_transcript_exon	-8.70562E-09	1.7	ENSSSCG00000043207 (2927), ENSSSCG00000043207 (1294)
WU_10.2_7_4900147	rs334407753	7	4794544	downstream	-8.70562E-09	1.7	ENSSSCG00000060969 (6708), ENSSSCG00000043136 (10312)
ASGA0030735	rs80851338	7	4838610	intergenic	-1.09902E-05	1.7	ENSSSCG00000043136 (35887), U6 (66874)
WU_10.2_7_4959871	rs333981734	7	4854266	intergenic	2.80021E-06	1.7	ENSSSCG00000043136 (51543), U6 (51218)
WU_10.2_7_4973517	rs80807606	7	4867925	intron	3.52873E-07	1.7	DSP
ALGA0038146	rs80786020	7	4883334	intron	-0.000857233	1.7	DSP
H3GA0019644	rs80971547	7	4915102	synonymous	-1.15643E-07	1.7	DSP
WU_10.2_7_5034795	rs327954472	7	4930409	intron	-5.25545E-08	1.7	SNRNP48
WU_10.2_7_5034795	rs327954472	7	4930409	intron	-5.25545E-08	1.7	SNRNP48 (11058), SNRNP48 (5627)
WU_10.2_7_5042138	rs80923163	7	4937752	downstream	5.43985E-06	1.7	SNRNP48 (18401), BMP6 (272518)
ASGA0030771	rs80830170	7	4950865	intergenic	-4.27339E-10	1.7	SNRNP48 (31514), BMP6 (259405)

WU_10.2_7_5102208	rs319172465	7	4997822	intergenic	-0.000146822	1.8	SNRNP48 (78471), BMP6 (212448)
M1GA0009455	rs80986401	7	5008671	intergenic	-5.68976E-05	1.8	SNRNP48 (89320), BMP6 (201599)
ASGA0030788	rs80792076	7	5031479	intergenic	1.57357E-06	1.8	SNRNP48 (112128), BMP6 (178791)
WU_10.2_7_5162412	rs343176224	7	5058020	upstream	1.32455E-06	1.8	SNRNP48 (138669), BMP6 (152250)
M1GA0009457	rs80938216	7	5089734	intron	-0.07553443	1.9	BMP6
ASGA0040427	rs81405706	8	135274760	3_prime_UTR	3.80499E-07	1.7	ENSSSCG00000009240
ALGA0050145	rs81405710	8	135285532	intron	5.60078E-06	1.7	COPS4
WU_10.2_8_144693654	rs345777825	8	135398502	intron	3.7104E-07	1.7	THAP9
ALGA0117619	rs81347425	8	135427306	intron	3.85982E-07	1.7	SEC31A
8_144744932	rs328673214	8	135449779	intron	5.65452E-07	1.7	SEC31A
WU_10.2_8_144771629	rs345296578	8	135476476	intron	1.85796E-06	1.7	SEC31A
WU_10.2_8_144847860	rs334436239	8	135487158	intron	4.3567E-07	1.7	SEC31A
H3GA0052926	rs81336088	8	135491186	intron	5.60382E-07	1.7	SEC31A
WU_10.2_8_144898378	rs319261432	8	135537530	intron	5.62168E-07	1.7	SCD5
WU_10.2_8_144927686	rs320260240	8	135566615	intron	3.87559E-06	1.7	SCD5
ALGA0121391	rs81328969	8	135591552	intron	3.27073E-09	1.7	SCD5
H3GA0054370	rs81328785	8	135604963	intron	6.01006E-08	1.7	SCD5
DRGA0017418	rs81339189	8	135607348	intron	3.69423E-08	1.7	SCD5
ALGA0109193	rs81337243	8	135629399	intron	3.68088E-08	1.7	SCD5
WU_10.2_8_145018548	rs338821365	8	135648560	downstream	9.09968E-08	1.7	ENSSSCG00000052420 (9892), ENSSSCG00000060705 (5196)
ASGA0082238	rs81330273	8	135665981	intergenic	2.52914E-07	1.7	ENSSSCG00000060705 (16178), U6 (54432)
ASGA0105760	rs81305967	8	135668712	intergenic	5.95358E-07	1.7	ENSSSCG00000060705 (18909), U6 (51701)
MARC0065298	rs81253423	8	135689152	intron	2.44483E-08	1.7	TMEM150C
ALGA0105830	rs339198846	8	135699278	intron	-0.000138345	1.7	TMEM150C
ASGA0092699	rs81311486	8	135719143	intron	-3.05086E-07	1.7	TMEM150C
ASGA0102835	rs81476910	8	135728719	intron	-3.04292E-07	1.7	TMEM150C
MARC0059365	rs81249045	8	135751832	intron	4.52085E-09	1.7	TMEM150C

ASGA0104181	rs81304104	8	135768303	downstream	0.02361438	1.7	U6 (47996), ENOPH1 (34085)
WU_10.2_10_5326729	rs331367368	10	3501675	downstream	1.00791E-07	1.7	BRINP3 (538148), ENSSSCG00000057470 (5265)
WU_10.2_10_5357268	rs340231888	10	3532219	intergenic	2.88977E-09	1.7	ENSSSCG00000057470 (29568), ENSSSCG00000053245 (65449)
WU_10.2_10_5369955	rs339644447	10	3544904	intergenic	1.12653E-09	1.7	ENSSSCG00000057470 (42253), ENSSSCG00000053245 (52764)
ALGA0056487	rs81425365	10	3572114	intergenic	0.001679716	1.7	ENSSSCG00000057470 (69463), ENSSSCG00000053245 (25554)
MARC0056124	rs81245706	10	3605230	intergenic	-0.001112193	1.7	ENSSSCG00000053245 (13082), ENSSSCG00000062242 (335879)
WU_10.2_10_5531412	rs328042345	10	3620686	intergenic	-0.000391314	1.7	ENSSSCG00000053245 (28538), ENSSSCG00000062242 (320423)
WU_10.2_10_5514795	rs330675368	10	3637304	intergenic	1.8132E-08	1.7	ENSSSCG00000053245 (45156), ENSSSCG00000062242 (303805)
MARC0078321	rs81263076	10	3649148	intergenic	0.002273651	1.7	ENSSSCG00000053245 (57000), ENSSSCG00000062242 (291961)
WU_10.2_10_5459685	rs333581856	10	3692446	intergenic	4.25061E-12	1.6	ENSSSCG00000053245 (100298), ENSSSCG00000062242 (248663)
WU_10.2_10_5624281	rs337718630	10	3827654	intergenic	8.8155E-09	1.6	ENSSSCG00000053245 (235506), ENSSSCG00000062242 (113455)
DRGA0010149	rs81302827	10	3843921	intergenic	8.83858E-09	1.6	ENSSSCG00000053245 (251773), ENSSSCG00000062242 (97188)
ALGA0056525	rs81426046	10	3937743	non_coding_transcript_exon	0.04626359	1.6	ENSSSCG00000062242
10007668	rs81218852	10	19391507	synonymous	-1.25652E-07	2.7	TLR5
H3GA0029533	rs81421707	10	19409070	intron	1.1656E-17	2.7	TLR5
CASI0009135	rs345690779	10	19411706	upstream	-3.33206E-07	2.7	TLR5 (24040), SUSD4 (140134)
ALGA0057679	rs81421752	10	19512266	intron	0.01626834	2.7	SUSD4
WU_10.2_10_22034045	rs344136854	10	19535825	intron	0.008806583	2.1	SUSD4
ASGA0046986	rs81421775	10	19572163	intergenic	-1.23299E-09	1.9	SUSD4 (150598), ENSSSCG00000010888 (14231)
WU_10.2_10_22164312	rs327591080	10	19604335	intergenic	-0.000786143	1.9	ENSSSCG00000010888 (19173), ENSSSCG00000054070 (54172)
WU_10.2_10_22123753	rs342419073	10	19645727	intergenic	-3.17315E-07	1.9	ENSSSCG00000010888 (60565), ENSSSCG00000054070 (12780)
MARC0010568	rs3474528732	10	19715055	intron	-9.68071E-08	1.9	CAPN8
ASGA0095681	rs81315152	10	19753037	intergenic	-1.06555E-07	1.9	CAPN8 (87182), CAPN2 (67209)
MARC0105091	rs3471802107	10	19780059	intron	0.02230717	1.9	CAPN2

ASGA0098449	rs81318702	10	24795288	intron	1.94965E-07	1.6	KDM5B
WU_10.2_10_29208538	rs346173073	10	24850398	intergenic	-0.03287021	1.6	ENSSSCG00000021179 (8097), ENSSSCG00000044728 (5116)
ASGA0084454	rs81343376	12	6971812	intergenic	3.78362E-05	3.7	RPL38 (86910), ENSSSCG00000046071 (647627)
MARC0075255	rs81260438	12	6982421	intergenic	1.65511E-11	3.7	RPL38 (97519), ENSSSCG00000046071 (637018)
M1GA0026980	rs81323860	12	6995769	intergenic	1.02398E-08	3.7	RPL38 (110867), ENSSSCG00000046071 (623670)
ALGA0103381	rs81478115	12	7006069	regulatory_region	7.43838E-12	3.7	RPL38 (121167), ENSSSCG00000046071 (613370)
ASGA0052681	rs81436220	12	7048858	intergenic	2.03123E-10	3.6	RPL38 (163956), ENSSSCG00000046071 (570581)
H3GA0033126	rs81436398	12	7067951	intergenic	2.62612E-05	3.6	RPL38 (183049), ENSSSCG00000046071 (551488)
MARC0046612	rs81236893	12	7080205	intergenic	1.37058E-10	3.6	RPL38 (195303), ENSSSCG00000046071 (539234)
WU_10.2_12_7124825	rs345556034	12	7097729	intergenic	1.82204E-07	3.6	RPL38 (212827), ENSSSCG00000046071 (521710)
H3GA0033141	rs81436583	12	7127237	intergenic	1.3506E-09	3.6	RPL38 (242335), ENSSSCG00000046071 (492202)
WU_10.2_12_7581758	rs337597858	12	7159210	intergenic	0.000174117	3.7	RPL38 (274308), ENSSSCG00000046071 (460229)
H3GA0033163	rs81436813	12	7188626	intergenic	-4.39043E-05	3.7	RPL38 (303724), ENSSSCG00000046071 (430813)
ALGA0064531	rs81436950	12	7225821	intergenic	-0.000206224	3.7	RPL38 (340919), ENSSSCG00000046071 (393618)
WU_10.2_12_7507773	rs326956108	12	7233173	intergenic	2.10104E-05	3.7	RPL38 (348271), ENSSSCG00000046071 (386266)
ASGA0052741	rs81437044	12	7247426	intergenic	-0.000223778	3.7	RPL38 (362524), ENSSSCG00000046071 (372013)
H3GA0033190	rs81437363	12	7283888	intergenic	2.79947E-08	3.7	RPL38 (398986), ENSSSCG00000046071 (335551)
DRGA0011569	rs81300895	12	7302134	intergenic	7.18764E-13	3.7	RPL38 (417232), ENSSSCG00000046071 (317305)
WU_10.2_12_7398515	rs81437611	12	7341458	intergenic	0.000697784	3.7	RPL38 (456556), ENSSSCG00000046071 (277981)
WU_10.2_12_7394362	rs325492649	12	7345606	intergenic	4.95795E-05	3.7	RPL38 (460704), ENSSSCG00000046071 (273833)
WU_10.2_12_7385966	rs318399475	12	7354001	intergenic	0.003297275	3.7	RPL38 (469099), ENSSSCG00000046071 (265438)
WU_10.2_12_7634052	rs338584089	12	7354388	intergenic	6.68669E-05	3.6	RPL38 (469486), ENSSSCG00000046071 (265051)
ASGA0090707	rs81309086	12	7371353	intergenic	0.01305383	3.6	RPL38 (486451), ENSSSCG00000046071 (248086)
ALGA0105065	rs81332319	12	7414272	intergenic	1.40866E-05	3	RPL38 (529370), ENSSSCG00000046071 (205167)
ASGA0095520	rs81314949	12	7418252	intergenic	0.000256823	3	RPL38 (533350), ENSSSCG00000046071 (201187)
ASGA0084859	rs335889358	12	7418384	regulatory_region	9.88463E-07	3	RPL38 (533482), ENSSSCG00000046071 (201055)
ASGA0083611	rs81338615	12	7420790	intergenic	1.144E-12	3	RPL38 (535888), ENSSSCG00000046071 (198649)

H3GA0054071	rs81325851	12	7421785	intergenic	0.000211604	3	RPL38 (536883), ENSSSCG00000046071 (197654)
ASGA0092609	rs81311382	12	7458764	intergenic	-3.38458E-07	3	RPL38 (573862), ENSSSCG00000046071 (160675)
M1GA0026970	rs81323725	12	7467740	intergenic	0.1113275	3.1	RPL38 (582838), ENSSSCG00000046071 (151699)
WU_10.2_15_135856391	rs337090786	15	122539331	intergenic	0.001690958	2.5	ENSSSCG00000062066 (111506), ENSSSCG00000060048 (12313)
ALGA0087403	rs81455161	15	122578140	intergenic	-0.01960898	2.5	ENSSSCG00000060048 (29911), ENSSSCG00000059125 (63084)
ASGA0070852	rs81455155	15	122598272	intergenic	4.44778E-12	1.7	ENSSSCG00000060048 (50043), ENSSSCG00000059125 (42952)
WU_10.2_15_135925574	rs324455174	15	122608399	intergenic	0.001238929	1.7	ENSSSCG00000060048 (60170), ENSSSCG00000059125 (32825)
WU_10.2_15_135980884	rs322071406	15	122663668	intergenic	0.001104923	1.7	ENSSSCG00000059125 (26755), EPHA4 (839458)
ALGA0087385	rs81455131	15	122682398	intergenic	3.21097E-08	1.6	ENSSSCG00000059125 (45485), EPHA4 (820728)
WU_10.2_15_136009793	rs81455128	15	122692545	intergenic	3.21097E-08	1.6	ENSSSCG00000059125 (55632), EPHA4 (810581)
ALGA0087381	rs81455126	15	122704300	intergenic	2.3191E-08	1.6	ENSSSCG00000059125 (67387), EPHA4 (798826)
H3GA0045110	rs81455122	15	122718113	intergenic	-0.00262943	1.6	ENSSSCG00000059125 (81200), EPHA4 (785013)
WU_10.2_15_136367391	rs345442617	15	122964587	intergenic	-2.84282E-10	1.6	ENSSSCG00000059125 (327674), EPHA4 (538539)
WU_10.2_15_136319027	rs340393759	15	123015411	intergenic	-7.63959E-10	1.7	ENSSSCG00000059125 (378498), EPHA4 (487715)
MARC0010057	rs81263210	15	123051624	intergenic	-0.08380099	1.7	ENSSSCG00000059125 (414711), EPHA4 (451502)
ALGA0106375	rs81333847	16	72782016	intron	-2.07868E-13	2.1	SEMA5A
ALGA0109858	rs81338026	16	72782953	intron	-1.25077E-09	2.1	SEMA5A
WU_10.2_16_78743591	rs341244976	16	72797915	intron	9.35678E-09	2.1	SEMA5A
WU_10.2_16_78747732	rs337368987	16	72802056	intron	9.5397E-10	2.1	SEMA5A
WU_10.2_16_78767897	rs323134864	16	72822331	intron	-8.0133E-11	1.9	SEMA5A
ALGA0106517	rs81334031	16	72831317	intron	-2.09489E-09	1.9	SEMA5A
WU_10.2_16_78796653	rs346390358	16	72852336	intron	-4.03912E-10	1.9	SEMA5A
ALGA0091889	rs81462644	16	72868185	intron	-1.80393E-09	1.9	SEMA5A
ASGA0074361	rs81462651	16	72889488	intron	1.06788E-05	2	SEMA5A
ASGA0105318	rs81305400	16	72895691	intron	-0.00101005	2	SEMA5A
ALGA0091895	rs81462668	16	72901318	intron	-0.001010387	1.9	SEMA5A

ASGA0074369	rs81462674	16	72959005	intron	-0.000894904	1.9	SEMA5A
WU_10.2_16_78907155	rs328025938	16	72978738	intron	-0.001082639	1.9	SEMA5A
ASGA0074372	rs81462678	16	72984010	intron	-0.000887138	1.9	SEMA5A
WU_10.2_16_78925811	rs319849366	16	72998820	intron	-9.383E-08	1.9	SEMA5A
WU_10.2_16_78946145	rs340236444	16	73019118	intron	0.000449775	1.9	SEMA5A
MARC0040336	rs81233287	16	73041036	intron	-0.000415253	1.9	SEMA5A
H3GA0047211	rs81462722	16	73056992	intron	-0.000100288	1.9	SEMA5A
H3GA0047209	rs81462697	16	73139057	intron	-0.000300235	1.9	SEMA5A
M1GA0021223	rs81462700	16	73153511	synonymous	-0.000764642	1.9	SEMA5A
WU_10.2_16_79204951	rs336130944	16	73236825	synonymous	-0.07519184	1.8	SEMA5A
ALGA0097014	rs81470637	18	11247266	intergenic	0.01582865	1.6	ENSSSCG00000050345 (134395), ENSSSCG00000044154 (57140)

[1]percentage of genetic variance explained by 0.52 Mb; [2]gene symbols when intronic or gene symbols (distance) adjacent to the marker when intergenic

Table S4 Summary of GWAS with the significant 0.52 Mb windows that were associated with the total number of stillbirth (TNS in Korean Berkshire pigs.

SNP	rsid	chr	Position	Variant	SNP effect	gVar(%)[1]	Gene annotation[2]
WU_10.2_1_282489432	rs338021506	1	251731634	intron	-5.29386E-07	1.9	MUSK
WU_10.2_1_282489432	rs338021506	1	251731634	intron	-5.29386E-07	1.9	MUSK (80924), MUSK (24974)
ASGA0093392	rs81474187	1	251748865	intron	3.09836E-13	2.9	MUSK
WU_10.2_1_282594005	rs332137984	1	251781867	intergenic	-0.000120496	3.4	MUSK (131157), ENSSSCG00000059916 (16593)
H3GA0055661	rs791879427	1	251816790	intron	-0.000114396	3.4	ENSSSCG00000005457
H3GA0055661	rs791879427	1	251816790	intron	-0.000114396	3.4	ENSSSCG00000005457 (5206), ENSSSCG00000044924 (14456)
MARC0062690	rs81251291	1	251824144	intron	0.000119609	3.4	ENSSSCG00000005457
ASGA0102913	rs81474268	1	251853287	intron	-2.65151E-11	3.3	ENSSSCG00000005457
ASGA0102913	rs81474268	1	251853287	intron	-2.65151E-11	3.3	ENSSSCG00000044924 (26296), ENSSSCG00000005457 (125872)
WU_10.2_1_282649558	rs334406549	1	251857701	intron	-2.0887E-12	3.3	ENSSSCG00000005457
WU_10.2_1_282662716	rs329545849	1	251870861	intron	-1.98214E-14	3.3	ENSSSCG00000005457
ASGA0006708	rs80863919	1	251939764	intron	5.84335E-14	3.3	ENSSSCG00000005457
