# Supplementary material for: Comprehensive genome-wide analysis of genetic loci and candidate genes associated with litter traits in purebred Berkshire pigs of Korea
Source: Anim Biosci. 2024 Aug 18;37(10):1702–11. doi: 10.5713/ab.24.0046 (PMC11366516; doi:10.5713/ab.24.0046)
Supplement: Supplementary file 4 [file ab-24-0046-Supplementary-Table-4.pdf]

ASGA0074369	rs81462674	16	72959005	intron	-0.000894904	1.9	SEMA5A
WU_10.2_16_78907155	rs328025938	16	72978738	intron	-0.001082639	1.9	SEMA5A
ASGA0074372	rs81462678	16	72984010	intron	-0.000887138	1.9	SEMA5A
WU_10.2_16_78925811	rs319849366	16	72998820	intron	-9.383E-08	1.9	SEMA5A
WU_10.2_16_78946145	rs340236444	16	73019118	intron	0.000449775	1.9	SEMA5A
MARC0040336	rs81233287	16	73041036	intron	-0.000415253	1.9	SEMA5A
H3GA0047211	rs81462722	16	73056992	intron	-0.000100288	1.9	SEMA5A
H3GA0047209	rs81462697	16	73139057	intron	-0.000300235	1.9	SEMA5A
M1GA0021223	rs81462700	16	73153511	synonymous	-0.000764642	1.9	SEMA5A
WU_10.2_16_79204951	rs336130944	16	73236825	synonymous	-0.07519184	1.8	SEMA5A
ALGA0097014	rs81470637	18	11247266	intergenic	0.01582865	1.6	ENSSSCG00000050345 (134395), ENSSSCG00000044154 (57140)

[1]percentage of genetic variance explained by 0.52 Mb; [2]gene symbols when intronic or gene symbols (distance) adjacent to the marker when intergenic

Table S4 Summary of GWAS with the significant 0.52 Mb windows that were associated with the total number of stillbirth (TNS in Korean Berkshire pigs.

SNP	rsid	chr	Position	Variant	SNP effect	gVar(%)[1]	Gene annotation[2]
WU_10.2_1_282489432	rs338021506	1	251731634	intron	-5.29386E-07	1.9	MUSK
WU_10.2_1_282489432	rs338021506	1	251731634	intron	-5.29386E-07	1.9	MUSK (80924), MUSK (24974)
ASGA0093392	rs81474187	1	251748865	intron	3.09836E-13	2.9	MUSK
WU_10.2_1_282594005	rs332137984	1	251781867	intergenic	-0.000120496	3.4	MUSK (131157), ENSSSCG00000059916 (16593)
H3GA0055661	rs791879427	1	251816790	intron	-0.000114396	3.4	ENSSSCG00000005457
H3GA0055661	rs791879427	1	251816790	intron	-0.000114396	3.4	ENSSSCG00000005457 (5206), ENSSSCG00000044924 (14456)
MARC0062690	rs81251291	1	251824144	intron	0.000119609	3.4	ENSSSCG00000005457
ASGA0102913	rs81474268	1	251853287	intron	-2.65151E-11	3.3	ENSSSCG00000005457
ASGA0102913	rs81474268	1	251853287	intron	-2.65151E-11	3.3	ENSSSCG00000044924 (26296), ENSSSCG00000005457 (125872)
WU_10.2_1_282649558	rs334406549	1	251857701	intron	-2.0887E-12	3.3	ENSSSCG00000005457
WU_10.2_1_282662716	rs329545849	1	251870861	intron	-1.98214E-14	3.3	ENSSSCG00000005457
ASGA0006708	rs80863919	1	251939764	intron	5.84335E-14	3.3	ENSSSCG00000005457

DRGA0002331	rs80855017	1	251969178	intron	4.29912E-14	3.3	ENSSSCG00000005457
DRGA0002331	rs80855017	1	251969178	intron	4.29912E-14	3.3	ENSSSCG00000044924 (142187), ENSSSCG00000005457 (9981)
ALGA0009238	rs80796469	1	252048165	intergenic	-4.90466E-05	3.3	ENSSSCG00000044924 (221174), OR2K2 (156888)
ALGA0009241	rs81351729	1	252065641	intergenic	0.000318225	3.3	ENSSSCG00000044924 (238650), OR2K2 (139412)
ASGA0006726	rs80837660	1	252080736	intergenic	-0.000290017	3.2	ENSSSCG00000044924 (253745), OR2K2 (124317)
WU_10.2_1_282945364	rs321619340	1	252114281	intergenic	-2.81677E-11	3.2	ENSSSCG00000044924 (287290), OR2K2 (90772)
H3GA0004278	rs80998992	1	252150964	intergenic	1.22452E-06	3.2	ENSSSCG00000044924 (323973), OR2K2 (54089)
MARC0021903	rs80890612	1	252166930	intergenic	4.92493E-05	3.2	ENSSSCG00000044924 (339939), OR2K2 (38123)
ASGA0006736	rs80843083	1	252179117	intergenic	-3.6569E-08	3.2	ENSSSCG00000044924 (352126), OR2K2 (25936)
ALGA0009256	rs80822720	1	252203530	downstream	0.004170362	3.2	ENSSSCG00000044924 (376539), OR2K2 (1523)
DIAS0002996	rs81212970	1	252226444	synonymous	0.004410882	1.8	ECPAS
WU_10.2_2_2746883	rs342277625	2	4093991	synonymous	0.000616272	3.1	TPCN2
ALGA0011405	rs3469852568	2	4134845	downstream	2.46251E-09	3	MRGPRF (11016), ENSSSCG00000055208 (7801)
ASGA0008471	rs3475937152	2	4163306	intergenic	-2.2799E-11	3	ENSSSCG00000042011 (8075), IGHMBP2 (31219)
WU_10.2_2_2770500	rs336307381	2	4190826	intron	0.003077523	3	IGHMBP2
MARC0011145	rs81256301	2	4209351	downstream	-8.01946E-12	2.3	ENSSSCG00000012879 (14781), CPT1A (84560)
WU_10.2_2_2807393	rs337343937	2	4226815	intergenic	-1.84254E-10	2.3	ENSSSCG00000012879 (32245), CPT1A (67096)
M1GA0002244	rs81362590	2	4267626	intron	-2.06779E-07	2.3	CPT1A
M1GA0002231	rs81362193	2	4279062	intron	8.8598E-06	2.3	CPT1A
M1GA0002231	rs81362193	2	4279062	intron	8.8598E-06	2.3	CPT1A (45963), CPT1A (14849)
MARC0070315	rs81256907	2	4291890	3_prime_UTR	0.000334255	2.3	CPT1A
M1GA0002229	rs81361514	2	4341271	downstream	6.71317E-06	2.2	TESMIN (43319), GAL (15376)
M1GA0002246	rs788180999	2	4365346	intergenic	-1.77015E-09	2.2	GAL (14233), PPP6R3 (158065)
H3GA0005584	rs81363333	2	4378975	intergenic	-1.54227E-15	2.2	GAL (27862), PPP6R3 (144436)
WU_10.2_2_2976035	rs336156132	2	4395779	3_prime_UTR	-4.0854E-15	2.2	PPP6R3
H3GA0005590	rs81364067	2	4412670	intron	-0.000209516	2.2	PPP6R3
WU_10.2_2_3004313	rs81364257	2	4424056	intron	0.000710609	2.2	PPP6R3

M1GA0002263	rs81364734	2	4444553	intron	-4.19525E-08	2.1	PPP6R3
M1GA0002265	rs81366488	2	4457240	intron	-1.67855E-08	2.1	PPP6R3
WU_10.2_2_3095872	rs328050650	2	4465980	intron	-7.50027E-06	2.1	PPP6R3
WU_10.2_2_3104145	rs341117829	2	4474254	intron	-0.000160581	2.1	PPP6R3
WU_10.2_2_3104145	rs341117829	2	4474254	intron	-0.000160581	2.1	PPP6R3 (79579), PPP6R3 (49157)
ALGA0109310	rs81337384	2	4531357	downstream	0.0019807	2	PPP6R3 (136682), LRP5 (122587)
2_3203612	rs325591449	2	4568691	intron	-1.12465E-11	1.8	LRP5
WU_10.2_2_3385614	rs326656297	2	4605668	intron	0.01364457	1.8	LRP5
WU_10.2_7_12567601	rs328248426	7	11971804	intergenic	0.002922849	4.8	ENSSSCG00000052001 (178986), ENSSSCG00000054172 (64218)
ALGA0038692	rs80898557	7	11989354	intergenic	9.00512E-05	4.2	ENSSSCG00000052001 (196536), ENSSSCG00000054172 (46668)
ALGA0038697	rs80852057	7	12045842	intergenic	5.83305E-05	4.2	ENSSSCG00000054172 (15545), ENSSSCG00000061595 (39182)
WU_10.2_7_12661047	rs341821663	7	12065247	intergenic	5.69232E-05	4.2	ENSSSCG00000054172 (34950), ENSSSCG00000061595 (19777)
H3GA0020000	rs80944404	7	12084107	non_coding_transcript_exon	5.69183E-05	4.2	ENSSSCG00000061595
H3GA0020000	rs80944404	7	12084107	non_coding_transcript_exon	5.69183E-05	4.2	ENSSSCG00000057253
WU_10.2_7_12695778	rs321301006	7	12099976	intergenic	5.69232E-05	4.6	ENSSSCG00000057253 (17515), MYLIP (37949)
ALGA0038703	rs80839173	7	12114493	upstream	-8.6652E-12	4.6	ENSSSCG00000057253 (32032), MYLIP (23432)
H3GA0020002	rs80900332	7	12135480	intron	-1.13184E-08	4.6	MYLIP
H3GA0020002	rs80900332	7	12135480	intron	-1.13184E-08	4.6	MYLIP (18240), MYLIP (2445)
DRGA0007138	rs80799020	7	12147428	downstream	1.5999E-12	4.6	MYLIP (30188), ENSSSCG00000051868 (10282)
ASGA0031290	rs80949504	7	12253352	upstream	-4.35925E-07	4.6	ENSSSCG00000057138 (19531), GMPR (46179)
H3GA0020006	rs80956848	7	12274672	intron	0.006182223	4.6	GMPR
ALGA0038729	rs80989281	7	12292990	intron	-2.21982E-05	3.1	GMPR
ALGA0038729	rs80989281	7	12292990	intron	-2.21982E-05	3.1	GMPR (38602), GMPR (6541)
WU_10.2_7_12911090	rs343319634	7	12312194	intron	-0.000666984	3.1	ATXN1
ALGA0038731	rs80889526	7	12319312	intron	-3.59692E-09	3.1	ATXN1
WU_10.2_7_12941421	rs341051420	7	12342523	intron	1.98613E-06	3.1	ATXN1

ASGA0031322	rs80826770	7	12424533	intron	-9.72746E-13	3.1	ATXN1
WU_10.2_7_13046917	rs338544958	7	12447284	intron	0.002268991	3.1	ATXN1
ALGA0038747	rs80865722	7	12471569	upstream	-0.01547467	2.6	ENSSSCG00000050012 (32833), ENSSSCG00000058629 (6005)
WU_10.2_7_123337647	rs326137282	7	116204637	intergenic	7.08337E-09	2.1	ENSSSCG00000052470 (63715), DICER1 (206587)
ALGA0045171	rs80842500	7	116217163	intergenic	-5.54189E-10	2.1	ENSSSCG00000052470 (76241), DICER1 (194061)
WU_10.2_7_123365158	rs330564343	7	116231882	intergenic	-2.36907E-10	2.1	ENSSSCG00000052470 (90960), DICER1 (179342)
WU_10.2_7_123428303	rs323468202	7	116245028	intergenic	-2.96718E-10	2.1	ENSSSCG00000052470 (104106), DICER1 (166196)
WU_10.2_7_123439493	rs325827236	7	116255475	intergenic	-3.13528E-10	2.1	ENSSSCG00000052470 (114553), DICER1 (155749)
WU_10.2_7_123510891	rs332486098	7	116267802	intergenic	-4.92004E-10	2.1	ENSSSCG00000052470 (126880), DICER1 (143422)
WU_10.2_7_123462504	rs321955839	7	116284431	intergenic	-4.57349E-10	2.1	ENSSSCG00000052470 (143509), DICER1 (126793)
DRGA0008201	rs81295354	7	116364906	downstream	-0.000457025	2.1	ENSSSCG00000052470 (223984), DICER1 (46318)
WU_10.2_7_123606943	rs332749698	7	116391085	intron	-0.00042139	2	DICER1
WU_10.2_7_123628307	rs344132124	7	116412448	upstream	-0.000383178	2	DICER1 (46646), ENSSSCG00000058870 (17959)
ASGA0036574	rs81396330	7	116448075	non_coding_transcript_exon	-6.31363E-05	1.9	ENSSSCG00000042615
MARC0096399	rs81275736	7	116458411	non_coding_transcript_exon	-0.003218372	1.9	ENSSSCG00000057191
WU_10.2_8_6092382	rs327739985	8	6710408	intron	5.97339E-14	3.8	CLNK
WU_10.2_8_6106489	rs333398011	8	6724514	intron	-9.07196E-07	3.8	CLNK
WU_10.2_8_6125875	rs319954748	8	6744237	intron	2.18373E-07	3.8	CLNK
WU_10.2_8_6146460	rs328690324	8	6764825	intron	9.10017E-10	3.8	CLNK
WU_10.2_8_6159481	rs81319995	8	6777847	intergenic	-2.36959E-10	3.8	CLNK (168994), ENSSSCG00000019781 (171784)
WU_10.2_8_6175644	rs344361676	8	6794010	intergenic	-1.41529E-12	3.8	CLNK (185157), ENSSSCG00000019781 (155621)
WU_10.2_8_6191745	rs331315480	8	6810112	intergenic	-5.7472E-10	3.8	CLNK (201259), ENSSSCG00000019781 (139519)
ASGA0102441	rs81323760	8	6828177	intergenic	-2.9748E-09	3.8	CLNK (219324), ENSSSCG00000019781 (121454)
ASGA0037686	rs81400507	8	6872552	intergenic	1.19216E-11	3.8	CLNK (263699), ENSSSCG00000019781 (77079)
WU_10.2_8_6265476	rs333531017	8	6883842	intergenic	0.003419204	3.8	CLNK (274989), ENSSSCG00000019781 (65789)
WU_10.2_8_6282086	rs328818294	8	6900448	intergenic	0.002124428	2.8	CLNK (291595), ENSSSCG00000019781 (49183)

ALGA0046317	rs81400484	8	6913947	intergenic	0.001154526	2.3	CLNK (305094), ENSSSCG00000019781 (35684)
WU_10.2_8_6311315	rs320630523	8	6929677	intergenic	0.003943262	2	CLNK (320824), ENSSSCG00000019781 (19954)
MARC0091202	rs81272275	8	111449788	intergenic	-2.76833E-05	5.9	ENSSSCG00000052805 (64800), ENSSSCG00000048102 (153834)
ASGA0087256	rs81476824	8	111464396	intergenic	2.62628E-06	5.9	ENSSSCG00000052805 (79408), ENSSSCG00000048102 (139226)
WU_10.2_8_119634232	rs339401102	8	111517851	intergenic	-3.04859E-10	5.9	ENSSSCG00000052805 (132863), ENSSSCG00000048102 (85771)
WU_10.2_8_119582517	rs325014808	8	111569348	intergenic	3.26187E-10	5.9	ENSSSCG00000052805 (184360), ENSSSCG00000048102 (34274)
ALGA0049192	rs80883173	8	111761621	intron	0.004913499	5.9	ENPEP
DIAS0002802	rs321087883	8	111782056	synonymous	0.004438352	3.9	ENPEP
ALGA0049202	rs81403466	8	111899793	intergenic	1.37156E-05	2.5	ENSSSCG00000061296 (22637), ENSSSCG00000059739 (61208)
H3GA0035082	rs81438393	12	59320767	intron	-0.001350146	1.8	NCOR1
ALGA0067282	rs81438375	12	59345524	intron	-0.001199853	2.8	NCOR1
ALGA0067281	rs81438369	12	59360809	intron	-0.001199766	2.5	NCOR1
MARC0022618	rs3473462527	12	59383668	intron	9.48567E-11	2.2	NCOR1
H3GA0035071	rs81438351	12	59392739	downstream	3.37503E-10	3.1	NCOR1 (119639), TTC19 (26256)
H3GA0056745	rs3471050222	12	59458957	intergenic	-0.001458141	3.1	ADORA2B (27444), SPECC1 (195235)
M1GA0025160	rs81346733	12	59461443	intergenic	0.001193354	2.7	ADORA2B (29930), SPECC1 (192749)
M1GA0024295	rs81332721	12	59464857	downstream	0.001929041	2.5	ADORA2B (33344), SPECC1 (189335)
ASGA0094328	rs81313474	12	59496739	intron	0.001226922	2.1	SPECC1
ASGA0091832	rs81310434	12	59503071	intron	-1.01791E-11	4.3	SPECC1
ASGA0082073	rs81329435	12	59539052	intron	1.46362E-08	4.3	SPECC1
MARC0104547	rs81280830	12	59541318	intron	6.64452E-05	4.3	SPECC1
M1GA0027152	rs81304684	12	59560760	intron	-7.34023E-14	4.3	SPECC1
H3GA0053129	rs81338548	12	59563268	intron	-2.4048E-15	4.3	SPECC1
ALGA0104388	rs3473880743	12	59677731	intergenic	0.00097769	4.3	SPECC1 (210940), AKAP10 (111400)
ALGA0107991	rs81335837	12	59749745	intron	2.79241E-13	3.9	AKAP10
ASGA0102677	rs81324047	12	59797489	intergenic	0.001789455	3.9	AKAP10 (65366), ULK2 (80899)

ALGA0114929	rs81344019	12	59813764	intron	0.002474634	3.3	ULK2
ALGA0101633	rs1110513197	12	59816448	intron	-7.20534E-09	2.4	ULK2
ALGA0123244	rs3475085828	12	59832996	intron	-9.96337E-08	2.4	ULK2
ASGA0092849	rs81311680	12	59841644	intron	0.002474634	2.4	ULK2
12_62758000	rs320765022	12	59850669	intron	0.002907074	1.7	ULK2
WU_10.2_16_67121359	rs330273501	16	61893496	intergenic	4.38028E-12	2.8	GABRA6 (38028), ENSSSCG00000041732 (84918)
WU_10.2_16_67160369	rs326989708	16	61932459	intergenic	-2.46419E-15	2.8	GABRA6 (76991), ENSSSCG00000041732 (45955)
WU_10.2_16_67192252	rs332293021	16	61964335	intergenic	-0.000234706	2.8	GABRA6 (108867), ENSSSCG00000041732 (14079)
MARC0012270	rs81274670	16	61996687	intron	-8.22578E-06	4.2	GABRB2
WU_10.2_16_67254880	rs341752931	16	62026615	intron	-0.000212155	4.2	GABRB2
ALGA0091166	rs81460896	16	62049121	intron	-4.12043E-09	4.1	GABRB2
WU_10.2_16_67294713	rs331465234	16	62066324	intron	0.007642582	4.1	GABRB2
H3GA0046863	rs80906125	16	62204054	intron	-0.001242286	1.6	GABRB2
H3GA0047915	rs81465288	17	12045367	intron	6.06369E-08	1.7	ENSSSCG00000007004
ASGA0075463	rs81465284	17	12062356	intron	3.74967E-13	2.2	ENSSSCG00000007004
WU_10.2_17_9746718	rs340492327	17	12078075	intron	-0.000139007	2.2	ENSSSCG00000007004
ALGA0093288	rs81465260	17	12101104	intergenic	0.000705847	2.2	ENSSSCG00000044174 (13118), ENSSSCG00000044124 (53238)
MARC0030380	rs81224989	17	12149145	intron	-0.000231872	2.4	ENSSSCG00000044124
ASGA0075444	rs81465216	17	12196160	downstream	-7.04026E-12	2.4	ENSSSCG00000054385 (17943), ENSSSCG00000057786 (4142)
H3GA0056000	rs81318643	17	12203390	non_coding_transcript_exon	-2.00149E-05	2.4	ENSSSCG00000050111
ALGA0103930	rs81330993	17	12208959	non_coding_transcript_exon	-0.000217364	2.4	ENSSSCG00000053036
WU_10.2_17_9948917	rs321561826	17	12231242	non_coding_transcript_exon	0.001147339	2.3	ENSSSCG00000050358
ALGA0093251	rs80805260	17	12246865	upstream	5.60891E-13	2	ENSSSCG00000053983 (9259), ENSSSCG00000049959 (28173)
MARC0073651	rs80990714	17	12267480	upstream	-0.000476289	2	ENSSSCG00000053983 (29874), ENSSSCG00000049959 (7558)
WU_10.2_17_10016145	rs330330568	17	12290972	upstream	-0.000346484	2	ENSSSCG00000049959 (21001), ENSSSCG00000043573 (6477)
ASGA0075438	rs80926773	17	12314493	non_coding_transcript_exon	-0.004434238	1.9	ENSSSCG00000053635

WU_10.2_17_47439537	rs341429033	17	41963261	intron	4.10218E-12	3.3	DHX35
H3GA0049067	rs80839500	17	41992643	intergenic	1.05071E-09	3.3	DHX35 (91969), ENSSSCG00000052330 (31817)
H3GA0049069	rs80947840	17	42004893	intergenic	1.05375E-08	3.3	DHX35 (104219), ENSSSCG00000052330 (19567)
ASGA0077089	rs80893851	17	42041944	intergenic	-3.22364E-10	3.3	ENSSSCG00000052330 (22816), ENSSSCG00000059132 (1171945)
WU_10.2_17_47551924	rs335477633	17	42050589	intergenic	9.17533E-09	3.3	ENSSSCG00000052330 (31461), ENSSSCG00000059132 (1163300)
ALGA0123517	rs81321389	17	42088366	intergenic	-4.54524E-11	3.3	ENSSSCG00000052330 (69238), ENSSSCG00000059132 (1125523)
ALGA0115746	rs81345071	17	42094505	intergenic	-4.27215E-10	3.3	ENSSSCG00000052330 (75377), ENSSSCG00000059132 (1119384)
WU_10.2_17_47607350	rs324518159	17	42106145	intergenic	4.55665E-14	3.3	ENSSSCG00000052330 (87017), ENSSSCG00000059132 (1107744)
MARC0030914	rs81223926	17	42139020	intergenic	-1.03371E-09	5.1	ENSSSCG00000052330 (119892), ENSSSCG00000059132 (1074869)
WU_10.2_17_47660553	rs329873968	17	42159212	intergenic	6.65328E-17	6.1	ENSSSCG00000052330 (140084), ENSSSCG00000059132 (1054677)
WU_10.2_17_47706743	rs328557833	17	42205215	intergenic	-0.001929919	6.2	ENSSSCG00000052330 (186087), ENSSSCG00000059132 (1008674)
WU_10.2_17_47726361	rs319285946	17	42224736	intergenic	-3.71419E-14	5.3	ENSSSCG00000052330 (205608), ENSSSCG00000059132 (989153)
WU_10.2_17_47838187	rs327193499	17	42337026	intergenic	-2.02618E-18	5.6	ENSSSCG00000052330 (317898), ENSSSCG00000059132 (876863)
ASGA0105916	rs81306152	17	42384529	intergenic	-0.002357563	5.6	ENSSSCG00000052330 (365401), ENSSSCG00000059132 (829360)
WU_10.2_17_47939784	rs321435842	17	42438628	intergenic	0.004743875	4.6	ENSSSCG00000052330 (419500), ENSSSCG00000059132 (775261)
WU_10.2_17_47961469	rs324568734	17	42460314	intergenic	-0.003761393	3.1	ENSSSCG00000052330 (441186), ENSSSCG00000059132 (753575)
MARC0046530	rs81237579	17	42475688	intergenic	-0.004767463	2	ENSSSCG00000052330 (456560), ENSSSCG00000059132 (738201)
MARC0043409	rs81236332	18	4068201	intergenic	4.40447E-07	2.1	DPP6 (919718), ENSSSCG00000042794 (461571)
WU_10.2_18_4338795	rs323156321	18	4146336	intergenic	-4.61669E-08	2.5	DPP6 (997853), ENSSSCG00000042794 (383436)
ASGA0104044	rs81303945	18	4195580	intergenic	5.45778E-06	2.5	DPP6 (1047097), ENSSSCG00000042794 (334192)
ASGA0100463	rs81321238	18	4224596	intergenic	-7.41321E-14	2.5	DPP6 (1076113), ENSSSCG00000042794 (305176)
WU_10.2_18_4437222	rs328805423	18	4246811	intergenic	4.92362E-10	2.5	DPP6 (1098328), ENSSSCG00000042794 (282961)
WU_10.2_18_4462931	rs344227291	18	4271505	intergenic	3.3585E-15	2.5	DPP6 (1123022), ENSSSCG00000042794 (258267)
WU_10.2_18_4485274	rs326167881	18	4295878	intergenic	0.000981217	2.5	DPP6 (1147395), ENSSSCG00000042794 (233894)

ASGA0082264	rs81479433	18	4317527	intergenic	-2.4243E-05	2.2	DPP6 (1169044), ENSSSCG00000042794 (212245)
WU_10.2_18_4519592	rs327464983	18	4330182	intergenic	1.18731E-10	2.2	DPP6 (1181699), ENSSSCG00000042794 (199590)
ASGA0099518	rs81320033	18	4366119	intergenic	1.02755E-05	2.2	DPP6 (1217636), ENSSSCG00000042794 (163653)
WU_10.2_18_4586954	rs341567333	18	4399265	intergenic	-0.001570039	2.2	DPP6 (1250782), ENSSSCG00000042794 (130507)
ASGA0090699	rs81309073	18	4433333	intergenic	-2.5336E-10	1.9	DPP6 (1284850), ENSSSCG00000042794 (96439)
ASGA0084020	rs81340828	18	4440434	intergenic	3.13944E-08	1.9	DPP6 (1291951), ENSSSCG00000042794 (89338)
ALGA0119307	rs81326437	18	4453405	intergenic	1.33326E-13	1.9	DPP6 (1304922), ENSSSCG00000042794 (76367)
H3GA0056437	rs81324290	18	4468276	intergenic	1.333E-13	1.9	DPP6 (1319793), ENSSSCG00000042794 (61496)
ASGA0095228	rs81314564	18	4469780	downstream	3.14292E-13	1.9	DPP6 (1321297), ENSSSCG00000042794 (59992)
MARC0044433	rs81236859	18	4474319	downstream	1.92406E-08	1.9	DPP6 (1325836), ENSSSCG00000042794 (55453)
WU_10.2_18_4691045	rs333666929	18	4502879	intron	8.12823E-09	1.9	ENSSSCG00000042794
ALGA0115679	rs81344984	18	4516477	intron	-1.84705E-08	1.9	ENSSSCG00000042794
ASGA0097549	rs81317538	18	4522341	intron	-1.69901E-08	1.9	ENSSSCG00000042794
ASGA0097549	rs81317538	18	4522341	intron	-1.69901E-08	1.9	ENSSSCG00000042794
WU_10.2_18_4727355	rs337165001	18	4538602	intergenic	0.002435025	1.9	ENSSSCG00000042794 (64108), ENSSSCG00000040865 (48677)

[1]percentage of genetic variance explained by 0.52 Mb; [2]gene symbols when intronic or gene symbols (distance) adjacent to the marker when intergenic
