# Supplementary material for: Comprehensive genome-wide analysis of genetic loci and candidate genes associated with litter traits in purebred Berkshire pigs of Korea
Source: Anim Biosci. 2024 Aug 18;37(10):1702–11. doi: 10.5713/ab.24.0046 (PMC11366516; doi:10.5713/ab.24.0046)
Supplement: Supplementary file 6 [file ab-24-0046-Supplementary-Fig-2.pdf]

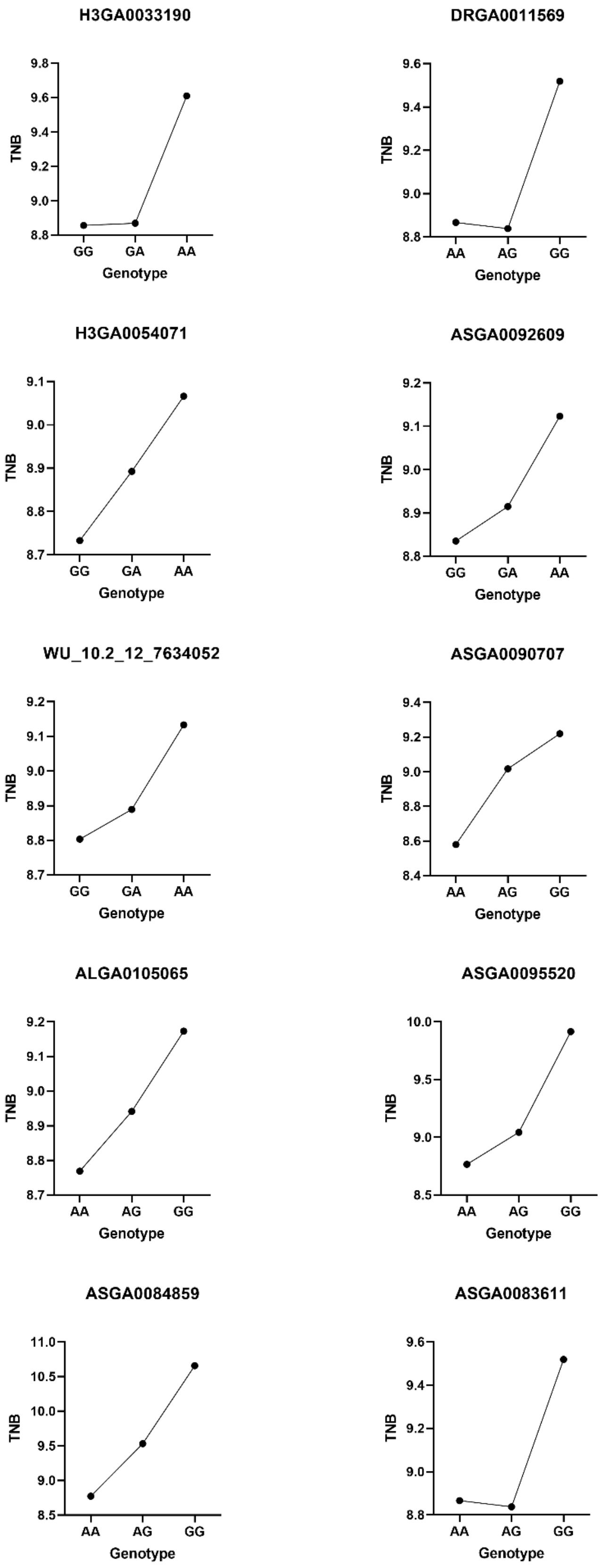

Fig S2. The phenotypic change according to the genotype of the markers that showed the highest genetic variance explained in NBA
